# A novel PDPN antagonist peptide CY12-RP2 inhibits melanoma growth via Wnt/β-catenin and modulates the immune cells

**DOI:** 10.1186/s13046-023-02910-y

**Published:** 2024-01-02

**Authors:** Chunyan Feng, Albert Yu, Zhongfu Wang, Kun Wang, Jiawei Chen, Yaojiong Wu, Ting Deng, Huaqing Chen, Yibo Hou, Shaohua Ma, Xiaoyong Dai, Laiqiang Huang

**Affiliations:** 1 Institute of Biopharmaceutical and Health Engineering, Shenzhen Key Laboratory of Gene and Antibody Therapy, State Key Laboratory of Chemical Oncogenomics, Shenzhen International Graduate School, Tsinghua University, Shenzhen, 518055 Guangdong China; 2 School of Life Sciences, Tsinghua University, Beijing, 100084 China; 3 Department of Interventional Radiology, Shenzhen People’s Hospital, 1017 Dongmen North Road, Shenzhen, 518020 NoGuangdong China

**Keywords:** Melanoma, PDPN, CY12-RP2 peptide, EMT, Wnt/β-catenin pathway, Immune activation

## Abstract

**Background:**

Podoplanin (PDPN) is a highly conserved, mucin-type protein specific to the lymphatic system. Overexpression of PDPN is associated with the progression of various solid tumors, and plays an important roles in the tumor microenvironment by regulating the immune system. However, the role of PDPN-mediated signal activation in the progression of melanoma is still unknown.

**Methods:**

PDPN expression was first analyzed in 112 human melanoma tissue microarrays and melanoma cell lines. Functional experiments including proliferation, clone formation, migration, and metastasis were utilized to identify the suppressive effects of PDPN. The Ph.D.TM-12 Phage Display Peptide Library was used to obtain a PDPN antagonist peptide, named CY12-RP2. The immunofluorescence, SPR assay, and flow cytometry were used to identify the binding specificity of CY12-RP2 with PDPN in melanoma cells. Functional and mechanistic assays in vivo and in vitro were performed for discriminating the antitumor and immune activation effects of CY12-RP2.

**Results:**

PDPN was overexpressed in melanoma tissue and cells, and inhibited melanoma cells proliferation, migration, and metastasis by blocking the EMT and Wnt/β-catenin pathway. PDPN antagonistic peptide, CY12-RP2, could specifically bind with PDPN, suppressing melanoma various functions inducing apoptosis in both melanoma cells and 3D spheroids. CY12-RP2 also enhanced the anti-tumor capacity of PBMC, and inhibited melanoma cells growth both in xenografts and allogeneic mice model. Moreover, CY12-RP2 could inhibit melanoma lung metastasis, and abrogated the immunosuppressive effects of PDPN by increasing the proportion of CD3 + CD4 + T cells, CD3 + CD8 + T cells, CD49b + Granzyme B + NK cells, and CD11b + CD86 + M1-like macrophages and the levels of IL-1β, TNF-α, and IFN-γ.

**Conclusions:**

This study has demonstrated the important role of PDPN in the progression of melanoma and formation of immunosuppressive environment, and provided a potential approach of treating melanoma using the novel CY12-RP2 peptide.

**Graphical Abstract:**

In melanoma, PDPN is overexpressed in the cancer cells, and promotes melanoma cells growth and metastasis through activating the Wnt/β-catenin pathway. Treatment with the PDPN antagonistic peptide CY12-RP2 could not only inhibit the melanoma growth and metastasis both *in vitro* and *in vivo* through Wnt/β-catenin pathway blockade, but also abrogate the immunosuppressive effects of PDPN through modulating immune cells.

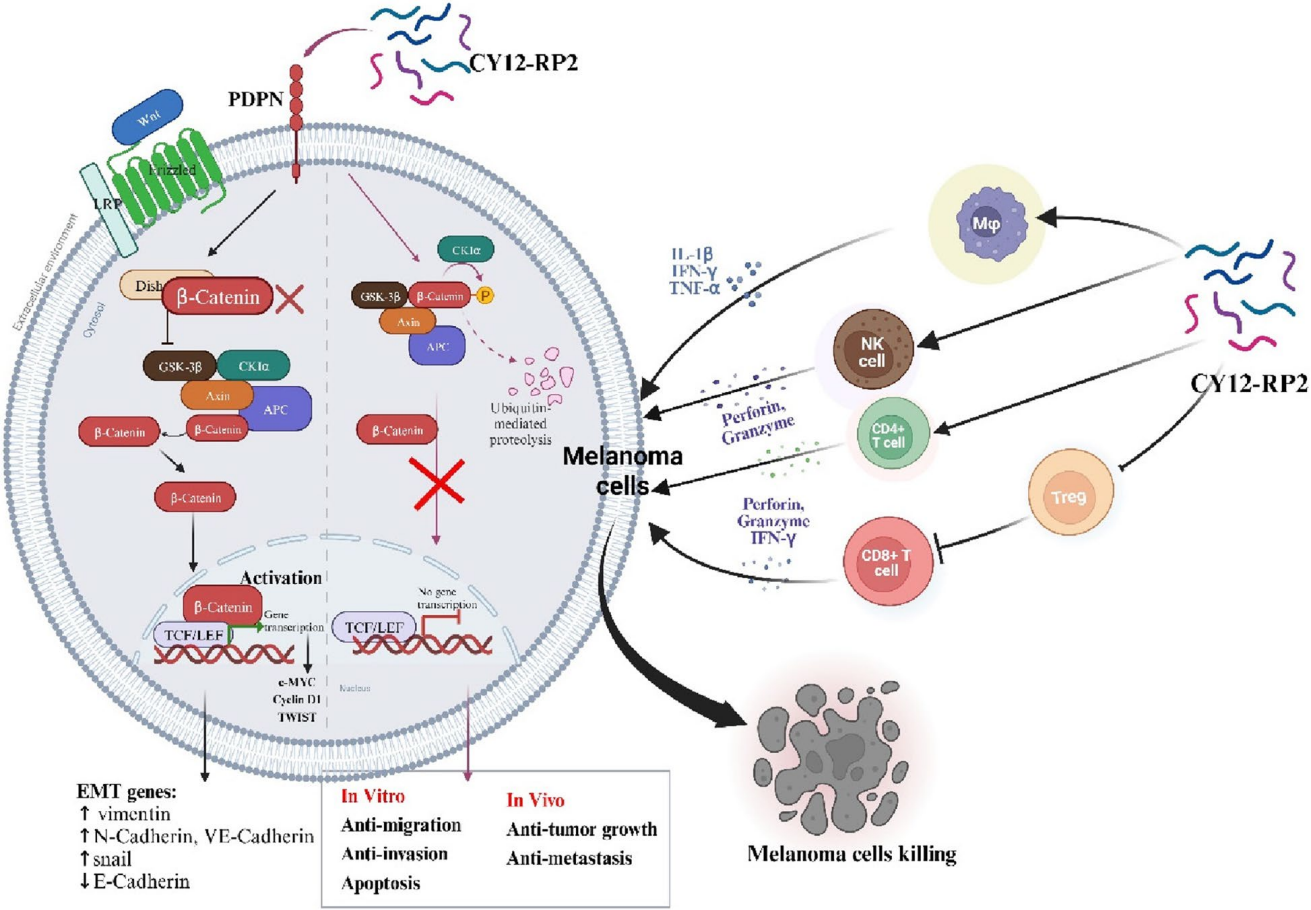

**Supplementary Information:**

The online version contains supplementary material available at 10.1186/s13046-023-02910-y.

## Background

Melanoma is a type of cutaneous cancer, and occurs predominantly due to the mutation and subsequent uncontrolled proliferation of pigment producing cells termed melanocytes [1, 2]. Although the incidence rate of melanoma is lower than other types of skin cancer, such as basal cell carcinoma and squamous cell carcinoma, melanoma is considerably more serious since it is more likely to metastasize when not diagnosed and treated in the early stages [3, 4]. Existing therapeutic options for melanoma include surgical excision of the lesion, radiation therapy, chemotherapy, immunotherapy, and targeted therapy [5, 6]. Among the treatment options, targeted therapy is extensively used, and involves inhibiting target proteins and related pathways involved in tumor proliferation, migration, invasion, and immunoevasion. Although targeted therapeutics such as the BRAF inhibitor vemurafenib, MEK inhibitor trametinib, and various PD-1/PD-L1 antibodies are approved for clinical use, the efficacy of these therapeutics are still limited by side effects and drug resistance [3, 7, 8]. It is urgent to explore and develop novel targeted therapeutics to improve the treatment of melanoma.

Podoplanin (PDPN) is an extremely conserved mucin-type protein, and exhibits high homology among various species [9, 10]. PDPN is a specific lymphatic endothelium marker, which is frequently expressed in some types of malignant tumors, including brain tumors, squamous cell carcinoma, and malignant mesothelioma [11, 12], and can be used as a diagnostic marker in cancer [13]. In addition, PDPN regulates cell proliferation, migration, invasion, epithelial-mesenchymal transition (EMT), and stemness associated pathways, all of which are critical for the tumor development [14]. A retrospective study demonstrated that 69.1% of 55 melanoma cases expressed PDPN, and over-expression of PDPN promoted cancer migration and EMT, thereby accelerating invasion and metastasis [15, 16]. Laura Bresson et al. has identified PDPN as regulator of Wnt/β-catenin signaling, and found that genetic deletion of PDPN affected the expression of Wnt/β-catenin signaling components in basal cells [17]. Nevertheless, the mechanism between PDPN and melanoma is still not clear and needs further elucidation. Additionally, tumor-expressed PDPN is the endogenous ligand of C-type lectin receptor (CLEC-2) [18], a transmembrane protein expressed on platelets that is involved in platelet activation and agglutination [19, 20]. The agglutinated platelets may wrap around the tumor cells and constitute a barrier between the tumor cells and immune cells to shield the tumor cells from the assault of immune cells [21, 22]. After platelets are conjugated with tumor cells, the activated platelets also secrete various cytokines into the tumor microenvironment to exert influence on the activities of tumor cells and immune cells [22–24]. Liu et al. found that tumor-infiltrating PDPN + cells (tPDPNs) could promote pro-tumor macrophage and dysfunctional CD8 + T cell infiltration to induce immunoevasive contexture in gastric cancer [25]. Hu et al. also found that PDPN was overexpressed in gastric cancer, positively correlated with immune cell infiltration levels, and closely correlated with immune markers of M2 type TAM and T cell exhaustion [26]. At present, research that focuses on the effects of PDPN on the melanoma tumor microenvironment is limited, and therapeutic peptides targeting PDPN for the prevention and/or treatment of melanoma have also not been reported.

Therapeutic peptides are a novel and promising type of anti-cancer drug, due to several important advantages over proteins or antibodies: they are molecularly small, easy to synthesize, and capable of penetrating cell membranes [27–30]; they also have high biological activity, affinity, chemical diversity, and specificity [31]. Phage display technology is a high-throughput screening technique that greatly amplifies the application of directional evolution techniques for antibody and peptide modification screening [32–34], and first described in 1985 by Smith. Phage display inserts the peptide sequence gene into the bacteriophage by using recombinant technology, and isolates the expressed peptides with the highest binding affinity by continuous elution. Phage display biopanning technique have shown promising applications in the field of cancer diagnosis and treatment. In this study, we obtained PDPN-targeted peptides by in vitro cell screening. Merits of in vitro cell biopanning include retaining the binding activities and biological functions, while identifying novel cell-surface receptors with unknown biological functions. Ting Deng et al. used the Ph.D.-12 phage display peptide library to obtain a novel FMOD antagonist peptide, labeled RP4, which could inhibit the growth of colorectal cancer through blocking the Akt and Wnt/β-catenin signaling pathways [35]. Kun Wang et al. has screened a novel FOXM1-targeting peptide from phage display library, and they then used the peptide to obtain p-PROTAC degrader of FOXM1, which induced degradation of FOXM1 protein and inhibited breast cancer and liver cancer growth [36]. Alisson L Matsuo et al. has reported that the [-CSSRTMHHC-] peptide, screened by C7C phage display library, could suppress melanoma growth and invasion both in vitro and in vivo. Therefore, using phage display to screen PDPN antagonist peptide may be a promising way to find novel therapeutics for melanoma targeted therapy [34].

In the current study, we firstly demonstrated that the overexpression of PDPN could promote the proliferation, morphology, migration, and invasion of melanoma through activating Wnt/β-catenin pathway. We then used the phage display peptide library to screen a high affinity and specificity peptide (CY12-RP2) targeting PDPN, and found that CY12-RP2 could not only inhibit the proliferation, migration, and invasion of melanoma both in vitro and in vivo, but also could suppress the melanoma growth via modulating the proportion of subpopulation of immune cells, including T cells, macrophages, and NK cells. Our results shed light on the importance of PDPN in melanoma progression, and also provided a novel PDPN-targeting peptide for melanoma treatment.

## Methods

### Cell culture and cell transfection

A375, A875, SK-MEL-28, B16-F10, and HEK293T cell lines were used in this research. The A375 and B16-F10 cell lines were obtained from Army Medical University (Chongqing, China). The A875, HEK293T, and SK-MEL-28 cell lines were purchased from Procell company (Wuhan, China). The A375, A875, B16F10, and HEK293T cells were cultured in DMEM medium (GIBCO, South America) supplemented with 10% FBS (GIBCO, South America), and 100 IU of penicillin and 100 mg/mL streptomycin (Beyotime, Shanghai, China). The SK-MEL-28 cells were cultured in RPMI1640 with 10% FBS and 100 IU of penicillin and 100 mg/mL streptomycin. All cells were incubated at 37 ℃ in a humidified atmosphere with 5% CO_2_. Lentivirus-mediated expression and RNA interference were used to establish stable clones for gene overexpression or knockdown of melanoma cells A375 or A875, respectively. Lentiviral particles were prepared with a PDPN-targeted knockdown sequence shRNA1: 5′-GCAGCTGACCTTAGGAC-3′. The PDPN cDNA fragments and negative control particles were purchased from Shangwei Biotechnology company (Shangwei, Shenzhen, China). Cells were seeded at a density of 5 × 10^5^ cells/well in the 6-well plates with complete medium, and the infection was performed when the cells were at 50% confluence. After co-incubating the cells with the harvested lentivirus for 6~8 h, the culture supernatant was substituted with a fresh medium. Stable cell lines were selected by incubating with 2 μg/mL of puromycin for 48 h after infection, and then puromycin-resistant stable cells were obtained.

### Peptide screening, design and synthesis

The Ph.D.TM-12 Phage Display Peptide Library Kit(~10^9^) (New England Biolabs) was used for Peptide screening following previously described prodecures [36]. In detail, HEK293T PDPN^+/+^ or HEK293T pcDNA stable cell lines were constructed through lentivirus infection. Lentivirus packaging vectors, PSPAX2 and VSVG, were co-transfected with target plasmid into HEK293T cells by UltraFection 3.0 transfection reagent (4A Biotech, Beijing, China) in a mass ratio of 2:1:3. Lentivirus were collected from cultured medium of HEK293T cells and enriched using lentivirus concentration solution (Yeasen, China) and centrifuged at 3000 g for 15 min to get lentivirus. HEK293T cells were infected by either control or PDPN overexpressed lentivirus, and selected with 2 μg/mL puromycin. Finally, puromycin-resistant stable PDPN overexpressed HEK293T cells or control cells were obtained for peptide screening. Stably transfected cell lines were cultured in 6-well plates (2 × 10^5^ cells/well) for 24 h. The phage display peptide libraries (10 μL) were incubated with HEK293T PDPN^+/+^ cells, and the unbound phages were removed by elution. Four rounds of elution were conducted to isolate the desired phage clones. Then, single-stranded DNAs (ssDNA) were obtained from the phage clones and sequenced by Biotech (Biotech, Shanghai, China). Finally, the peptides were synthesized by Biotech (Qiang Yao, Shanghai, China), and the purity of the peptides were confirmed to be 95% by high-performance liquid chromatography (HPLC) and mass spectrometry (MS).

### Real-time reverse transcription-quantitative PCR (RT-PCR) assays

Total RNA was isolated from melanoma cell lines by utilizing TRIzol reagent (Invitrogen, Thermo Fisher Scientific, USA) following the procedure of the manufacturer. Extracted RNA samples were reversely transcribed into cDNA using PrimeScript™ RT Master Mix (Perfect Real Time) (Takara, Shiga, Japan), and the quantitative real-time PCR was performed using the SYBR RT-PCR kit (Takara, Shiga, Japan), followed by quantitative RT-PCR technique to estimate relative transcript abundances. The level of PDPN was normalized to the endogenous control GAPDH. Expression levels of genes were calculated by performing the 2-ΔΔCq method. The primer sequences used in this experiment were as follows: PDPN (forward 5′-CGAAGATGATGTGGTGACTC-3′; reverse 5′-CGATGCGAATGCCTGTTAC-3′). MYC (forward 5′-AACAGAAAUGUCCUGAGCAAUTT-3′; reverse 5′-AUUGCUCAGGACAUUUCUGUUTT-3′). CCND1 (forward 5′-CCGTCCATGCGGAAGATC-3′; reverse 5′-ATGGCCAGCGGGAAGAC-3′). TWIST (forward 5′-GTCCGCAGTCTTACGAGGAG-3′; reverse 5′-GCTTGAGGGTCTGAATCTTGCT-3′). GAPDH (forward 5′-ACAGTCAGCCGCATCTTCTT-3′; reverse 5′-GACAAGCTTCCCGTTCTCAG-3′).

### Cell proliferation assay

Melanoma cell lines (A375 and A875) and transfected melanoma cells (A875 PDPN^+/+^ and A375 PDPN^−/−^) were seeded in 96-well plates at a density of 5 × 10^3^ cells/well for 24 h or 48 h. Cell proliferation assay was evaluated by using CCK8 reagent (Yeasen, Shanghai, China) according to the manufacturer’s protocols. Briefly, 10 μL of CCK8 solution was added to each well, and after thorough mixing the cells were further incubated for an additional 2~3 h. The absorbance was evaluated using the microplate reader (Biotek) at a wavelength of 450 nm.

### Measurement of cell viability

Cell viability was determined using a CCK8 kit. Melanoma cells were plated into 96-well plates at a density of 5 × 10^3^ cells/well. The cells were then treated with peptides at the determined concentrations for 24, 48, and 72 h. The CCK-8 (10 μL/ well) was pipetted into each well and the cells were incubated at 37 °C for 1~3 h. The viable cells were calculated by performing absorbance readings at 450 nm using a microplate reader (BioTek).

### 3D melanoma cells spheroids fabrication

A375 and A875 cells were collected and suspended in Matrigel (Corning) with a density of 1.0 × 10^7^ cells per mL. 3D spheroids were fabricate using cell-processing system that have two pumps. One pump was loaded with Matrigel and another with fluorocarbon oil (3 M NOVEC, HFE-7000). The flow rate of Matrigel was 20 μL/min and the fluorocarbon oil rate was 30 μL/min. The 3D spheroids were incubated at 37 °C with 5% CO_2_ after printing. Culturing medium was DMEM medium supplemented with 10% FBS, and 100 IU of penicillin and 100 mg/mL streptomycin. The medium was changed every 3 days. Afterward, the 3D spheroids were harvested for drug analysis.

### Study of the effect of CY12-RP2 on melanoma cell spheroids

Prepared melanoma cells spheroids were plated in 48-well plates. Following incubation for 3 days, the spheroids were exposed to different concentrations (0, 50, 100 and 200 μM) of CY12-RP2, and changes in spheroid diameter were detected after 5 days of treatment. Additionally, melanoma cell spheroids were treated with various concentrations of CY12-RP2 as described previously, and after 72 h, the spheroids were stained for immunofluorescence with caspase 3 primary antibody. The specific experimental procedures refer to the following immunofluorescence analysis. Additionally, we co-cultured peptide-stimulated PBMC with spheroids to determine the tumor-killing ability of activated PBMC.

### Wound-scratch assay

A375 GFP, A875 GFP, A375 PDPN^−/−^, and A875 PDPN^+/+^ cells were all seeded in 12-well plates at a density of 5 × 10^5^ cells/well. When the cells reached 100% confluence the next day, the straight wound was scraped in each cultured well with a 20 µL pipette tip. Then the cells were washed repeatedly with complete DMEM to remove the cellular debris, and the medium was changed to a low serum medium containing 0.05% FBS. The scratch-treated cells were subsequently cultured for an additional indicated time and imaged at 0 h, 12 h, 24 h and 48 h. Quantification of wound closure was accomplished by measuring the distance between the phase contrast images’ anterior edges of the contralateral surfaces using ImageJ software. As for peptide (CY12-RP2) function experiments, images of phase contrast for A375 GFP and A875 GFP cells were captured at 0 h before peptide administration. The cells were then treated with CY12-RP2 (0 μM, 5 μM, 25 μM) for 24 h or 48 h, and then images were acquired again. Handling of the experimental results were carried out as previously described.

### Matrigel invasion assay

For invasion assay, matrigel (Corning, New York, USA) was coated on the upper surface of the upper chamber. The upper chamber was filled with DMEM medium containing 2% FBS, while the lower chamber contained a medium supplemented with 20% FBS. In brief, melanoma cells (A375 GFP, A875 GFP, A375 PDPN^−/−^, and A875 PDPN^+/+^ cells) were seeded in the upper chamber of the transwell plate (Corning, New York, USA) at about 5 × 10^4^ cells/well with the medium as described above. For peptide functionality experiments, A375 and A875 cells were seeded in the upper chamber of the transwell plate at a density of 5 × 10^4^ cells/well with various peptide concentrations (0 μM, 5 μM, 25 μM). Transwell inserts were fixed using 4% paraformaldehyde after 48 h cell invasion, and crystalline violet was used for staining. The quantities of invading cells were quantified using ImageJ software (NIH).

### Western blotting assay

Melanoma cells were cultured in a 60 mm dish with a complete medium and various peptide concentrations (0 μM, 5 μM, 25 μM). Protein was extracted after cells were cultured or treated for 48 h using protein lysis buffer, separated using a 10% or 15% Bis–Tris PAGE gel (Epizyme Biomedical Technology, Shanghai, China), and transferred onto a PVDF membrane (Thermo Fisher Scientific) according to standard techniques. The primary antibodies used in this study were shown in the Table 1. The Western blots were imaged using Bio-rad Chemidoc MP and quantified using ImageJ software (NIH).

### Immunofluorescence analysis

Immunofluorescence analysis was used for antigen localization and quantification. For PDPN immunofluorescence staining, melanoma cells (A375, A875) were seeded in coverslips, cultured for 24 h, and fixed using 4% paraformaldehyde for 15 min at room temperature (RT). The cells were then permeabilized with 0.2% Triton X-100 in PBS for 10 min. Cells were blocked using 1% bovine serum albumin (BSA) in PBS for 30 min at RT, and incubated with the primary antibody overnight at 4 ℃. Afterwards, cells were washed three times with PBS containing 0.01% Triton X-100 for 5 min, and incubated with Alexa Fluor 568–conjugated secondary antibodies at RT for 1 h. Lastly, the nuclei were dyed with 4’,6-diamidino-2-phenylindole (DAPI) for 5 min at RT, and the slides were imaged using a NIKON laser scanning confocal microscope. To further verify the specificity binding ability of CY12-RP2 with PDPN, immunofluorescence was detected for analysis in melanoma cells. Briefly, A375 or A875 cells were incubated with CY12-RP2 at a concentration of 5 μM for 10 h at 37 ℃, and then washed with DMEM to remove the unbound peptide. The anti-PDPN primary antibody was then incubated for 4 h at RT. Further experimental procedures were performed as described above. To determine the immune cell infiltration and proportion changes in the lungs and spleens in the pulmonary metastasis mouse model, sequential frozen slices (12 μm) were prepared and incubated with CD3, CD4, CD8, Foxp3, CD56, F4/80, and CD11c primary antibodies for 1 h, and then incubated with Alexa Fluor 488-conjugated goat anti-mouse F(ab’)2 and Alexa Fluor 647-conjugated goat anti-mouse F(ab’)2 in PBS containing 1% BSA and 1% goat serum for 30 min. All incubations were conducted under coverslips at RT, and then repeatedly washed 3 times with PBS. Coverslips were mounted after staining cell nuclei with DAPI, and imaged using a NIKON laser scanning confocal microscope.

### Flow cytometry analysis

In this study, cell apoptosis was analyzed by flow cytometry using a PI/FITC-Annexin V Apoptosis Detection Kit (4A Biotech, Beijing, China). Briefly, melanoma cells were collected and washed with pre-chilled PBS, then resuspended with Annexin V staining buffer. Afterward, fluorescein isothiocyanate (FITC)-Annexin V and PI were stained for 5 min at RT, and then immediately detected using a Beckman flow cytometer. To investigate immune cell infiltration of the lung and spleen in the pulmonary metastasis mouse model, the surface and nuclear antigen staining were performed following the experimental procedure. Fluorescence-labeled mAb against CD3, CD25, CD49b, GranzymeB, CD11b, CD86, and CD206 were purchased from BioLegend (BioLegend, San Diego, CA), and CD4, CD8, and Foxp3 from Tonbo. Surface markers, including CD3, CD4, CD8, CD25, CD49b, GranzymeB, CD11b, CD86 and CD206, were firstly stained, then fixed, permeabilized, and stained for the intranuclear marker Foxp3, respectively. Finally, the samples were measured using flow cytometry.

### SPR assay

SPR assay was conducted on a GE Biacore 3000 (Cytiva, USA) using a CM7 sensorchip for protein immobilization. All experimental steps were performed at a temperature of 24 °C and samples were kept at 4 °C. CY12-RP1 and CY12-RP2 peptides were diluted in the running buffer (heparin-buffered saline buffer, HBS-EP) at the final concentrations ranging from 100 μM to 0.00128 μM. The peptides were injected at a rate of 30 μL/min through immobilized PDPN for 240 s for association. All regenerations were introduced at a flow rate of 30 µL/min. SPR assay was performed in triplicate and the results were analyzed using Biacore 3000 software.

### PBMC function assay

PBMCs (Shenzhen People's Hospital. Shenzhen, China)) from a healthy 38-year-old male donor were isolated using a density gradient centrifugation procedure. Firstly, the immune cell proportions analysis of PBMCs was measured using flow cytometry. We then investigated the effect of CY12-RP2 on the cytokines in PBMCs using ELISA assays. Briefly, PBMCs (5 × 10^5^ cells/well) were cultured in 24-well plates with RPMI1640 containing 10% FBS for 24 h, and the cells were stimulated with CY12-RP2 (50 µM) for 48 h. Afterward, supernatants were collected for subsequent assays. In addition, in order to demonstrate the killing capability of stimulated PBMCs on tumor cells, melanoma cells were co-cultured with stimulated PBMCs in various ratios (0%, 5%, 10%, 20%, 40%, and 50%). The experimental results were then observed by crystal violet staining after 48 h.

### Enzyme-linked immunosorbent assay (ELISA)

The levels of major inflammatory cytokines, including TNF-α, IL-1β, IL-2, TGF-β, IFN-γ, and IL-10, were measured using an ELISA kit (Shenzhen Ziker Biological Technology, Shenzen, China). Briefly, culture supernatants of cells and serum of pulmonary metastasis mouse model were respectively collected and added to an antibody pre-coated 96-well plates, and then followed up according to the manufacturer's instructions.

### In vivo biodistribution of peptide

In vivo biodistribution assay was conducted in BALB/c nude mice with melanoma tumor. Here we prepared 15 female BALB/c mice with subcutaneous melanoma tumor, and divided into three groups with each group of five. Mice were treated with His-CY12-RP2 by tail vein administration at various doses (25 mg/kg and 100 mg/kg). We collected at least 50 μL orbital blood samples by using capillary blood vessels at 6 h, 12 h, 24 h, 36 h, and 48 h, respectively. Five mice were used for the study of in vivo biodistribution of peptide. The organs including heart, liver, spleen, lung and kidney were collected after BALB/c nude mice were sacrificed at 48 h. The biodistribution of CY12-RP2 in blood and organs were measured using an ELISA kit (Shenzhen Ziker Biological Technology, Shenzhen, China) following the kit’s instructions.

### Bioluminescence imaging

Bioluminescence imaging was conducted using the IVIS Imaging System (Xenogen products from Caliper Life Sciences, Hopkinton, Massachusetts, USA) following the manufacturer's instructions. As for systemic bioluminescence, 100 μL D-luciferin substrate (5 mg/mL) was intraperitoneally administered roughly 10 min before imaging. Subsequently, mice were anesthetized with 1% isoflurane via intraperitoneal injection, and then used to acquire bioluminescence images. The exposure time varied from 2 to 5 min based on the luminescence signals of the injected cells. Imaging and quantization of the signals were performed using analysis software, and the quantization data was presented as a total number of photons/second.

### In vivo tumor growth and metastasis assays

Tumorigenesis assays were conducted in vivo using BALB/c nude mice and BALB/c mice from Guangdong Medical Laboratory Animal Center. The animal study was reviewed and approved by the Administrative Committee on Animal Research of Shenzhen International Graduate School, Tsinghua University (Ethical Development No. 16, 10 November 2021). Samples with 5 × 10^5^ A375 or B16F10-Luc melanoma cells were diluted in 100 μL PBS and injected subcutaneously into 6- to 8-week-old female BALB/c nude mice or BALB/c mice. 5 days later, the mice were treated with CY12-RP2 by tail vein administration at various doses (25 mg/kg and 100 mg/kg) every two days. The tumor volume and body weight were measured every 2 days, with the calculation of the tumor volume under the assumption of an ellipsoidal tumor as: tumor volume = length × width^2^/2. Tumors from BALB/c nude mice were collected and photographed on day 21, while tumors from the allograft mouse model were measured using bioluminescence imaging. The blood collected from the eyes of BALB/c nude mice were stored at 4 °C for 24 h and then centrifuged for 15 min under the indicated conditions (4 °C, 3000 rpm). The supernatant was collected and assayed for drug toxicity using kits, including AST, ALT, TG, and γ-GT.

For pulmonary metastasis assays, 6- to 8-week-old female BALB/c mice were administered with 5 × 10^5^ B16F10-Luc cells via tail vein to establish an experimental pulmonary metastasis model of melanoma. CY12-RP2 was administered to the mice intravenously at high and low doses (25 mg/kg and 100 mg/kg) every two days, and the mice were sacrificed after 15 days of treatment. Pulmonary metastases were evaluated using bioluminescence imaging on the 2^nd^, 5^th^, 10^th^, 15^th^ and 20^th^ day after B16F10-Luc cells injection. Subsequently, the BALB/c mice were sacrificed, and the organs including the heart, liver, spleen, lung, and kidney were collected and imaged for evaluation of micrometastases. The xenografts and organs were kept in a 4% paraformaldehyde solution for subsequent histological assays.

### Immunohistochemistry and histological analysis

For histopathological analysis, after BALB/c nude mice were sacrificed, organs (heart, liver, spleen, lung, and kidney) were collected and fixed in 4% paraformaldehyde for 24 h at RT. The fixed organs were then dehydrated and inserted in paraffin, and prepared in 2 µm sections. The prepared sections and experimental procedures were routinely carried out by Servicebio company.

### Statistical analysis

All statistical graphs in the article were plotted in GraphPad Prism 9, and the results were shown as mean ± standard deviation. In addition, we quantified the number and distance of cells by using ImageJ software. *P*-values used for determining statistical significance in the histograms were performed by the two-tailed student's t-test. The level of statistical significance was well-set at ∗ , *p* < 0.05; ∗  ∗ , *p* < 0.005; ∗  ∗  ∗ , *p* < 0.001; n.s. = not significant.

## Results

### PDPN is upregulated in melanoma, which is associated with the proliferation and metastasis of melanoma

**Fig. 1:**
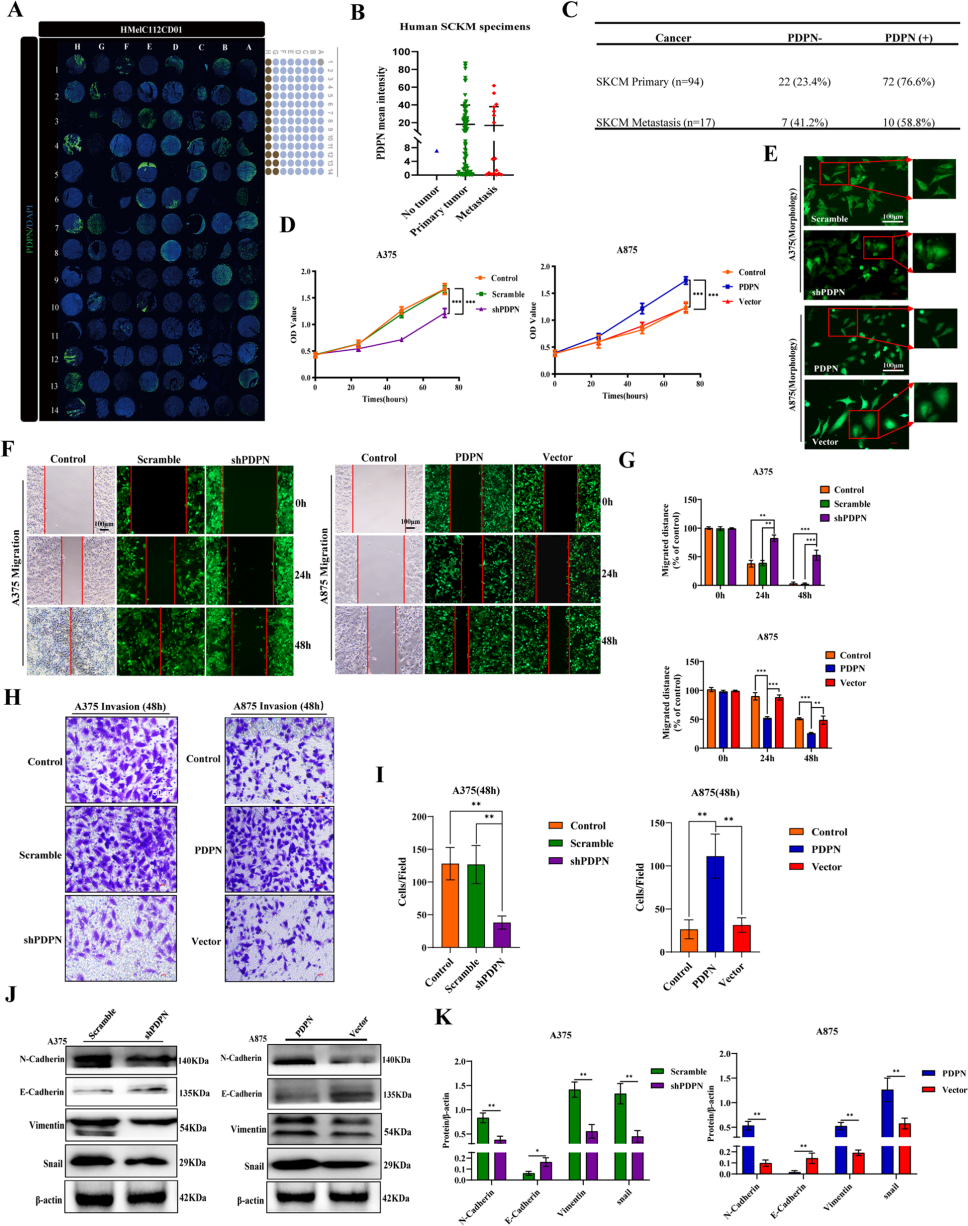
PDPN is upregulated in melanoma, and is associated with the proliferation and metastasis of melanoma. **A** Human melanoma tissue microarrays named HMelC112CD01 consisting of nontumor melanoma sample (*n* = 1), primary melanoma samples (*n* = 94), and melanoma distant metastasis samples (*n* = 17). **B** Human melanoma samples were subjected to immunofluorescence for PDPN with quantitative analyses. **C** Statistically PDPN expression with different melanoma samples. **D** PDPN expression correlated with melanoma cell lines (A375, A875) proliferation as measured by CCK8 assay. **E** Images demonstrated morphological changes after stable knockdown of PDPN in A375 cells and high expression of PDPN in A875 cells. The power field scale bar, 100 μm. **F**, **G** Wound healing assays were performed for the migration capability of A375 cells stably knockdown PDPN or A875 cells with PDPN overexpression, and statistical analysis was performed to determine the migrated distance. The power field scale bar, 100 μm. **H**, **I** Transwell analysis was performed to quantify the invasive ability of PDPN knockdown or overexpression cells, and statistical analysis was performed to determine the invasion of cells. The power field scale bar, 100 μm. **J**, **K** Western blot analysis was performed to identify the effects of PDPN on the EMT markers E-Cadherin, N-Cadherin, vimentin, and snail. β-actin as an internal control was used. The statistical analysis was performed to quantify the relative protein levels. Data are presented as mean ± SD. *p < 0.05, **p < 0.01, ***p < 0.001 versus control

PDPN is known for its broad range of functions, one of which entails involvement in tumorigenesis and metastasis [13]. Through analysis of the TCGA database, we found that PDPN was significantly associated with the development of several common types of tumors, including adenomas and adenocarcinomas, cystic, mucinous, and serous neoplasm, ductal and lobular neoplasia, and melanoma (Fig. S1A). To investigate the role of PDPN in the pathogenesis of melanoma, protein expression of PDPN was analyzed by using 112 human melanoma tissue microarrays named HMelC112CD01 consisting of nontumor melanoma sample (*n* = 1), primary melanoma samples (*n* = 94), and melanoma distant metastasis samples (*n* = 17) (Fig. 1A). The results showed that PDPN protein levels were dramatically upregulated in primary melanoma specimens and metastasis samples (Fig. 1B). According to the statistics, we found that 73.9% of 111 melanoma cases expressed PDPN (76.6% in 94 primary samples and 58.8% in 17 metastasis samples) (Fig. 1C). Then, we investigated the viability of PDPN expression in melanoma as a prognostic marker through the OncoLnc database, and found that high PDPN expression in clinical patients predicted a poor survival rate (Fig. S1B). Collectively, these results revealed that PDPN may be strongly associated with the formation and development of melanoma. We then examined the mRNA and protein expression levels of PDPN in melanoma cell lines (A375, A875, SK-MEL-28) by real-time RT-PCR and western blot, and found that PDPN was expressed variably in different melanoma cell lines (Fig. S1C and D). Furthermore, immunofluorescence also verified the differential expression of PDPN in melanoma cell lines (Fig. S1E). Thus, we chose the high PDPN expression cell line A375 and low expression cell line A875 for further studies. To investigate the effects of PDPN on melanoma cells, we used RNA interference to knockdown the PDPN in A375, and used overexpression lentiviral vectors to upregulate the PDPN in A875. As shown in Fig. S1F and G, the Western blot assays validated the expected regulation of PDPN both in A375 and A875 cells.

To identify the regulatory function of PDPN on the proliferation of melanoma cells, CCK8 assay was performed. The results showed that knockdown of PDPN (Lenti-shPDPN) in A375 cells led to a significant suppression of proliferation, while overexpression of PDPN (Lenti-PDPN) promoted the proliferation of A875 cells (Fig. 1D). In addition, morphological changes were also observed. The morphology of A375 cells changed from a square epithelial to a spindle-shaped mesenchymal phenotype when PDPN was down-regulated, while the morphology of A875 cells changed from rhombic to rounded phenotype when PDPN was up-regulated (Fig. 1E), which suggested that the expression level of PDPN could affect melanoma cells through morphological change. Moreover, the wound-healing and transwell assays revealed that the migration and invasion were significantly inhibited by Lenti-shPDPN in A375 cells, while the overexpression of PDPN could significantly promote the migration and invasion of A875 cells (Fig. 1F-I). EMT is a critical process for cancer cell metastasis [37]. As shown in Fig. 1J, K and Fig. S1H, the expression levels of proteins positively related to metastasis and invasion such as N-cadherin, VE-Cadherin, vimentin, and snail all significantly decreased while E-cadherin increased in A375 cells after PDPN knockdown. In contrast, the expression levels of N-cadherin, VE-Cadherin, vimentin, and snail all increased while E-cadherin decreased in A875 cells with induced PDPN overexpression. Taken together, these data demonstrated that PDPN is overexpressed in melanoma, closely correlated with poor prognosis, and play facilitating roles in melanoma cell proliferation, metastasis, and invasion.

### PDPN promotes melanoma metastasis via Wnt/β-catenin signaling pathway

**Fig. 2:**
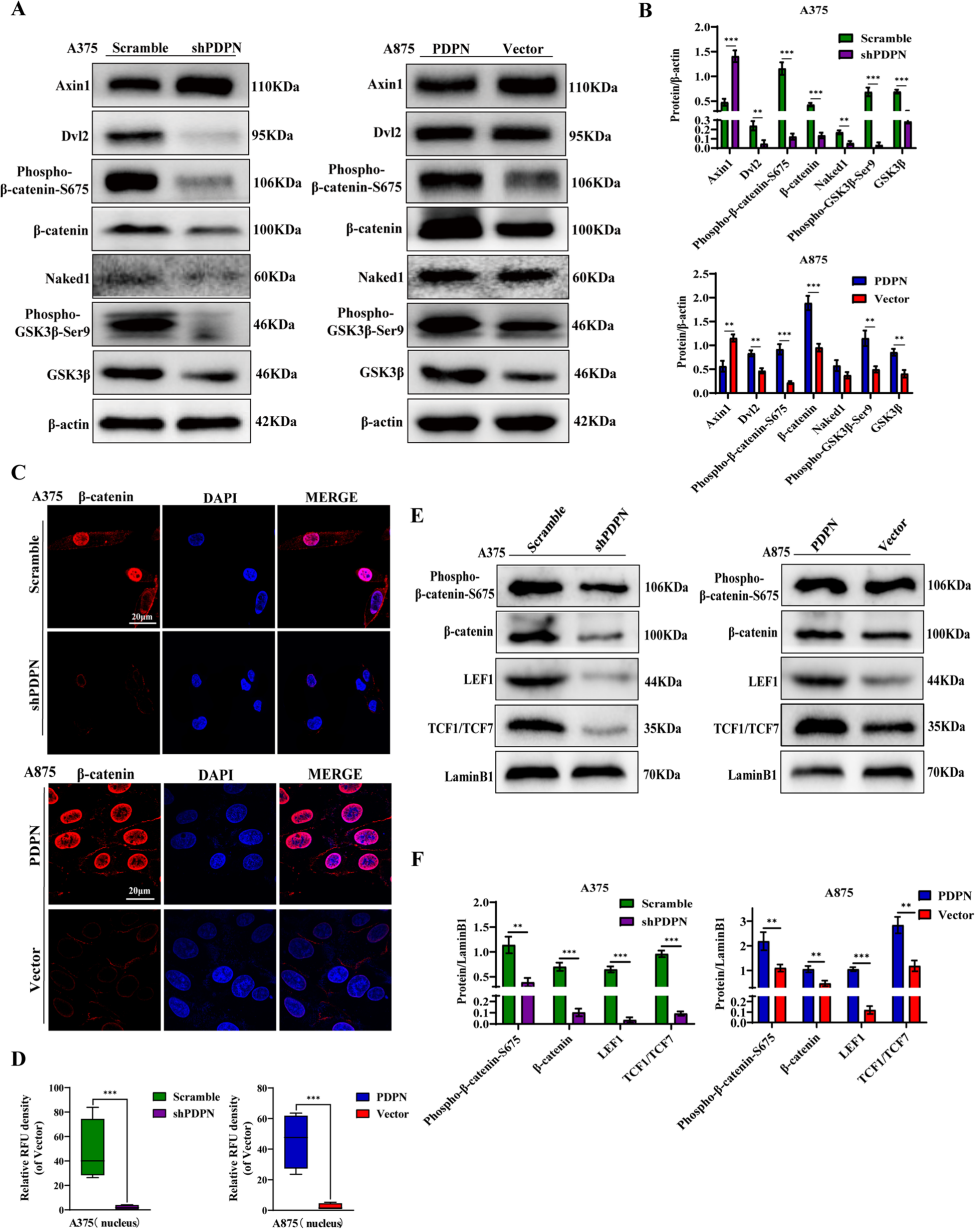
Effect of PDPN on the Wnt/β-Catenin signaling pathway in melanoma cells. **A** Western blot analysis was performed to detect the Wnt/β-catenin signaling-related proteins. β-actin as an internal control was used. **B** Statistical analysis was performed to quantify the relative protein levels. **C** Immunofluorescence staining was conducted to quantify PDPN effects on the nuclear β-catenin intensity in melanoma cell lines. The power field scale bar, 20 μm. **D** Statistical analysis was performed to quantify the immunofluorescence intensity of nuclear β-catenin in A875 cells. **E** Western blot assay was also performed on nuclear proteins related to the Wnt/β-catenin signaling pathway including β-catenin, phospho-β-catenin, LEF1, and TCF1/TCF7. The LaminB1 was used as an internal control for nuclear proteins. **F** Statistical analysis was performed to quantify the relative protein levels. Data are presented as mean ± SD. *p < 0.05, **p < 0.01, ***p < 0.001 versus control

The Wnt/β-catenin signaling is an important pathway of EMT, which is involved in the migratory and invasive properties of cancer cells [37]. To investigate the effect of PDPN on Wnt/β-catenin signaling pathway, the related proteins were quantified by Western blot. As shown in Fig. 2A and B, the results revealed that the protein levels of Dvl2, phospho-β-catenin-S675, β-catenin, Naked1, phospho-GSK3β-Ser9, and GSK3β were all significantly decreased, while Axin1 was increased in A375 cells with PDPN knockdown. Conversely, inducing PDPN overexpression activated the Wnt/β-catenin signaling pathway in A875 cells. In addition, we confirmed the expression of β-catenin by immunofluorescence analysis, and the results revealed that β-catenin expression was significantly decreased in PDPN knockdown A375 cells, while increased in PDPN overexpression A875 cells (Fig. 2C). Furthermore, as shown in Fig. 2E and F, the levels of β-catenin, phospho-β-catenin-S675, LEF1, and TCF1/TCF7 were all significantly down-regulated in the PDPN knockdown A375 cell nucleus, and conversely all significantly up-regulated in PDPN overexpression A875 cell nucleus. To further understand the mechanistic effects of PDPN, we detected both mRNA and protein expression of targeted genes of β-catenin, including c-MYC, Cyclin D1 and TWIST. The results showed that the mRNA levels of c-MYC, Cyclin D1 and TWIST were all significantly down-regulated in the PDPN knockdown A375 cells, and conversely all significantly up-regulated in PDPN overexpression A875 cells (Fig. S1G). To confirm the above mRNA results, we also detected protein levels of c-MYC, Cyclin D1 and TWIST. The levels of c-MYC, Cyclin D1 and TWIST were all significantly decreased in PDPN knockdown A375 cells, while increased in PDPN overexpression A875 cells (Fig. S1 H, I).

To confirm whether PDPN could affect migration and invasion of melanoma cells in wnt/β-catenin signaling pathway-dependent manner, A375 cells with PDPN knockdown were treated with a Wnt/β-catenin signaling pathway agonist (SKL2001) and PDPN overexpression A875 cells were treated with an inhibitor (IWR1) [38]. Wound-healing assays revealed that the inhibition of migration capacity in A375 cells by PDPN knockdown could be abolished by SKL2001, while the promotion of migration capacity in A875 cells by PDPN overexpression could be inhibited by IWR1 (Fig. S2A and B). Likewise, transwell assays indicated that the decrease or increase of invasion capacity in A375 or A875 cells mediated by PDPN were all abrogated by SKL2001 or IWR1, respectively (Fig. S2C and D). Furthermore, the levels of EMT positively correlated proteins, including N-cadherin, vimentin and snail were all suppressed, while E-cadherin was increased in A375 cells transfected with LV-shPDPN, and these phenomena were reversed following treatment with SKL2001 (Fig. S2E). In contrast, in the A875 cells, the activation of EMT by PDPN overexpression was blocked by IWR1 (Fig. S2E). Overall, these results indicated that PDPN could induce the EMT process in melanoma cells via activating the Wnt/β-catenin signaling pathway.

### Biopanning of PDPN antagonist peptides

**Fig. 3:**
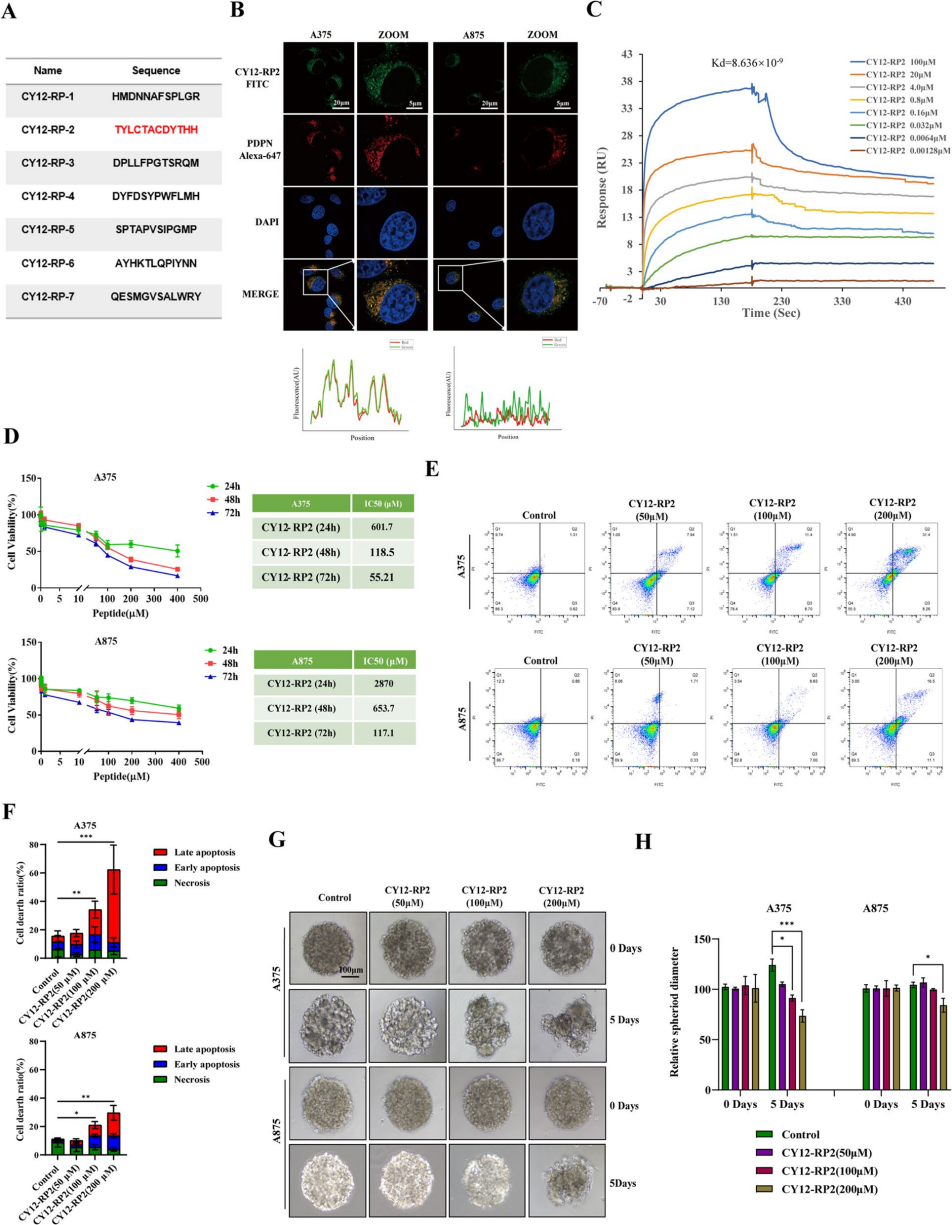
Biopanning of PDPN antagonist peptides. **A **A summary table of peptide sequences selected from the fourth round phage biopanning of PDPN peptides using a phage display library kit. **B** Immunofluorescence staining was performed to quantify the colocalization of CY12-RP2 and PDPN protein in melanoma cell lines (A375, A875). The power field scale bar, 20 μm. **C** CY12-RP2 (100, 20, 4.0, 0.8, 0.16, 0.032, 0.0064, 0.00128 μM) dose-dependent binding to PDPN. **D** Suppression efficiency of melanoma cells growth by CY12-RP2 was measured by CCK8 assay at various concentrations for 24, 48 or 72 h, respectively. **E**, **F** The A375 and A875 cells were treated with various concentrations of CY12-RP2 (0, 50, 100, 200 μM) for 48 h and analyzed using the Annexin V/PI staining flow cytometry (**E**), and statistical analysis was performed to quantify the apoptosis rates (**F**). **G**, **H** The 3D cellular spheres were treated with the set concentrations of CY12-RP2 for 5 days and cell morphology was assessed (**G**), relative spheroid diameter was measured (**H**). Data are presented as mean ± SD. *p < 0.05, **p < 0.01, ***p < 0.001 versus control. The power field scale bar, 100 μm

To further investigate the potential anti-tumor effect of PDPN antagonist on melanoma, we used a Ph.D.TM-12 Phage Display Peptide Library to screen for a PDPN antagonist peptide. After four rounds of biopanning under progressively stringent selection conditions in vitro, seven clones with individual amino acid sequences were obtained (Fig. 3A). To assess the anti-tumor effects of these seven peptides on melanoma cells, the proliferation of A375 and A875 cell lines after treating with various concentration peptides were examined by CCK8 assay. As shown in Fig. 3D and Fig. S3A, CY12-RP1 and CY12-RP2 dramatically inhibited cell growth in a dose-dependent manner in the PDPN overexpressed A375 cells, while having a relatively diminished impact on the PDPN underexpressed A875 cells. We then performed immunofluorescence, SPR assay, and flow cytometry to identify the binding specificity of CY12-RP1 and CY12-RP2 with PDPN in melanoma cells. As shown in Fig. 3B, CY12-RP2 showed a strong fluorescence and co-localization in PDPN overexpressed A375 cells and minimal fluorescence in PDPN underexpressed A875 cells, suggesting that the binding of CY12-RP2 to the cells depends on the expression level of PDPN. CY12-RP1 showed a weak fluorescence in both A375 and A875 cells (Fig. S3B), which indicated that the binding ability of CY12-RP2 was greater than CY12-RP1. Therefore, CY12-RP2 was chosen for further studies as the PDPN antagonist peptide. To further ascertain the direct interactions between CY12-RP2 and PDPN, we utilized SPR assay to investigate the binding affinity of CY12-RP2 for PDPN. PDPN recombinant proteins were coated on the CM7 sensor chip and peptide (CY12-RP1 and CY12-RP2) was circulated through. The results of SPR assay showed that CY12-RP2 bound to PDPN in a dose-dependent manner (Fig. 3C), whereas binding of CY12-RP1 to PDPN was minimal affected (Fig. S3C). The calculated Kd values of CY12-RP1 and CY12-RP2 for binding to PDPN were 4.592 × 10^–8^ and 8.636 × 10^–9^, respectively. These results indicated that CY12-RP2 could specifically bind to PDPN, allowing for the suppression of the development of melanoma.

Furthermore, flow cytometry assay was performed to investigate the apoptotic effect of CY12-RP2 on melanoma cells, with results showing that the apoptosis rate of A375 cells was significantly up-regulated by CY12-RP2 in a dose-dependent manner, while the increase in apoptosis rate in A875 cells was milder (Fig. 3E and F). The three-dimensional cellular sphere is a superior model for assaying the cytotoxicity of CY12-RP2 in melanoma cells. In this study, 3D melanoma cell spheroids were established using a cell-processing system, and representative images of 2D cells and 3D cellular spheres were shown in Fig. S3F. Subsequently, the 3D cell spheroids were used to verify the anti-tumor effects of CY12-RP2 at set concentrations (0, 50, 100 and 200 μM). The results showed that the relative A375 cellular spheroid diameter was significantly diminished with increasing CY12-RP2 concentration, while only a high concentration of CY12-RP2 (200 μM) could decrease the diameter of A875 cellular spheroids (Fig. 3G and H). To further investigate the apoptotic effect of CY12-RP2 on 3D melanoma cell spheroids, immunofluorescence staining was performed. The immunofluorescence staining showed that the level of Caspase3 was significantly increased by CY12-RP2 (Fig. S3G). Taken together, these results indicated that CY12-RP2 could specifically bind with PDPN, inhibit the cell proliferation, and promote apoptosis in melanoma cells both in 2D cells cultures and 3D cellular spheres.

### CY12-RP2 suppresses the invasion and migration of melanoma cells via blocking Wnt/β-catenin pathway

**Fig. 4:**
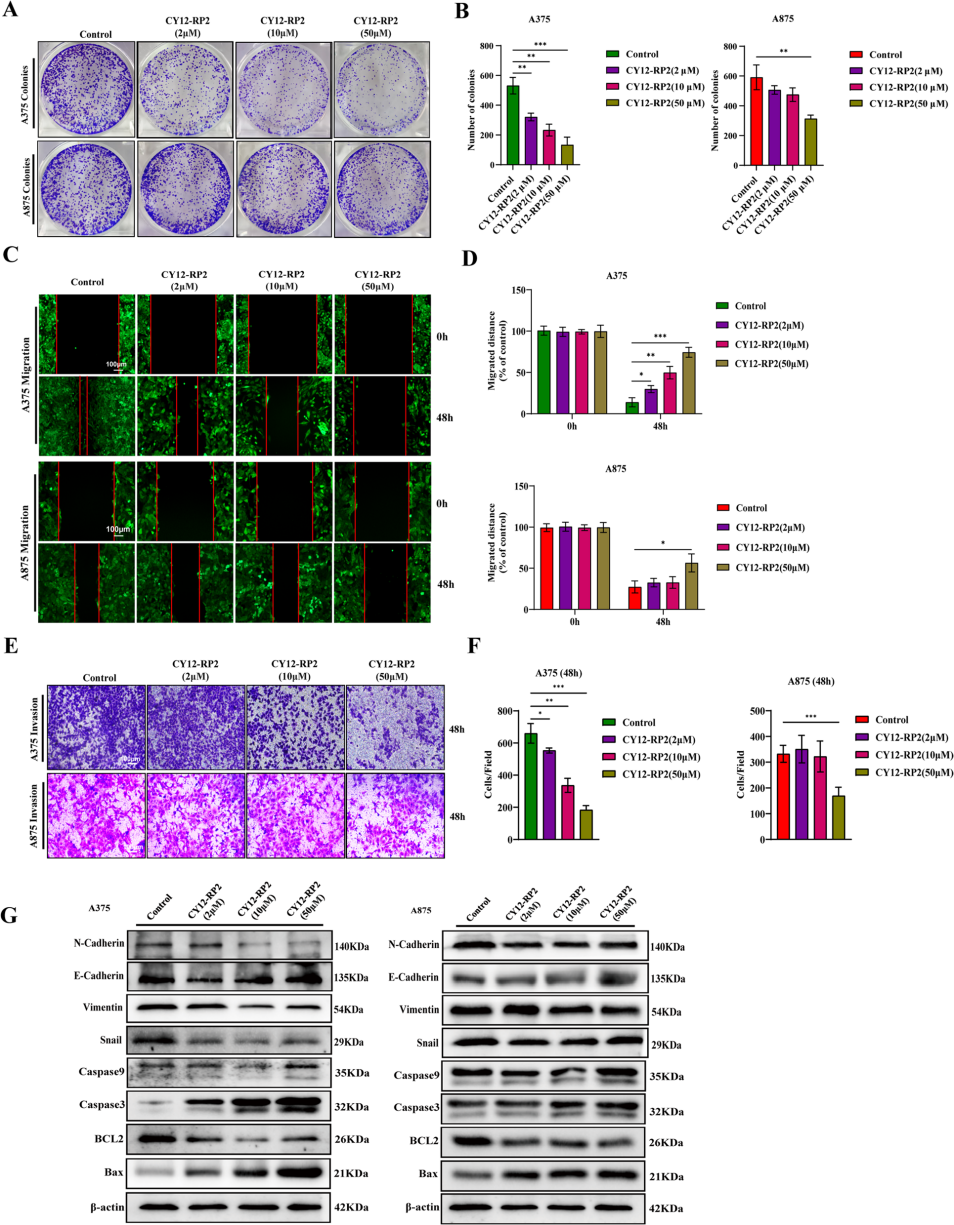
CY12-RP2 suppresses the invasion and migration of melanoma cells via blocking Wnt/β-catenin pathway. **A** The effect of CY12-RP2 on the colony formation of melanoma cell lines. **B** The colony formation statistical analysis. **C** Effects of CY12-RP2 on the migratory capacity of melanoma cell lines were analyzed by wound healing assay. The power field scale bar, 100 μm. **D** Statistical analysis was performed to determine the migrated distance. **E** Effects of CY12-RP2 on the invasive ability of melanoma cell lines were analyzed by transwell assays. The power field scale bar, 100 μm. **F** Statistical analysis was performed to determine the number of invaded cells. **G** The analysis of CY12-RP2 on EMT and apoptosis signaling pathways by using Western blot assay. Data are presented as mean ± SD. *p < 0.05, **p < 0.01, ***p < 0.001 versus control

We have demonstrated that PDPN was strongly associated with the proliferation, migration, and invasive properties of melanoma cells. Thus, the effects of CY12-RP2 on colony formation, metastasis and invasion in melanoma cell lines (A375, A875) were studied. As shown in Fig. 4A and B, CY12-RP2 could significantly suppress the colony formation of A375 cells in a dose-dependent manner, while only having inhibitory effects on A875 cells at high concentrations. Results of the wound healing assay demonstrated that compared with the control group, administration of the set concentrations of CY12-RP2 inhibited migration at the corresponding time both in A375 and A875 cells, but A375 cells were more susceptible (Fig. 4C and D). Furthermore, CY12-RP2 significantly suppressed the invasion of A375 cells in a dose-dependent manner, while the invasion of A875 cells could only be inhibited at high concentrations (Fig. 4E and F). The Western blot results showed that the expression of N-cadherin, vimentin, snail, and BCL2 all decreased, while expression of E-cadherin, Caspase-9, Caspase-3, and Bax all significantly increased in A375 cells after treatment with CY12-RP2. A similar trend in expression levels were also observed in A875 cells, but less significant when compared with A375 cells (Fig. 4G). In order to verify whether CY12-RP2 could specifically inhibit the migration and invasion of melanoma cells through Wnt/β-catenin signaling pathway, an agonist of the Wnt/β-catenin pathway, SKL2001, was used. The results showed that SKL2001 significantly reversed the inhibitory effect of CY12-RP2 on melanoma cell migration (Fig. S4A and B) and invasion (Fig. S4C and D). Additionally, Western blotting analysis indicated that SKL2001 significantly reversed the inhibitory effect of CY12-RP2 on EMT and Wnt/β-catenin signaling pathway (Fig. S4E). These results demonstrated that CY12-RP2 inhibited the migration and invasion of melanoma cells by blocking the Wnt/β-catenin signaling pathway.

### CY12-RP2 inhibits the growth of melanoma cells xenografts in vivo

**Fig. 5:**
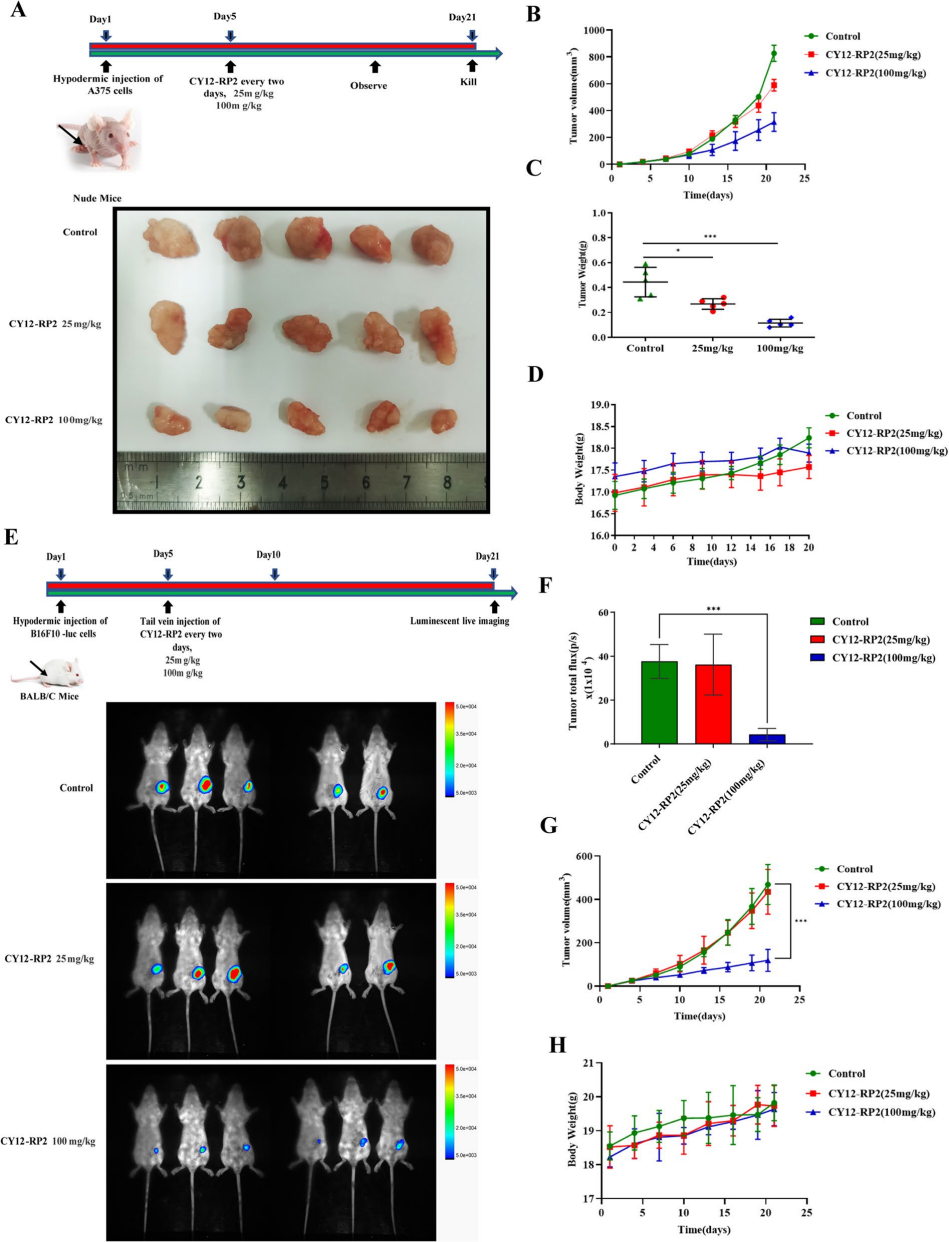
Effects of CY12-RP2 on melanoma tumorigenesis in vivo. **A** Melanoma cell line A375 was subcutaneously implanted in BALB/c nude mice, and 5 days later treated with CY12-RP2 (25 mg/kg, 100 mg/kg) every two day for 15 days. Tumor mass was resected after the 15-day treatment period. **B** Tumor growth curve of subcutaneous melanoma xenograft. **C** The weight of orthotopic xenografts tumors at the end of the experiment. **D** Body weight of BALB/c nude mice over the course of the experiment. **E** Murine melanoma cell line B16F10-luc was subcutaneously implanted in BALB/c nude mice, and 5 days later treated with CY12-RP2 (25 mg/kg, 100 mg/kg) every two day for 15 days. Bioluminescence imaging of tumor growth in BALB/c mice was performed. **F** Statistical analysis of bioluminescence imaging. **G** The tumor volume after treating with CY12-RP2. **H** The body weight of mice after treating with CY12-RP2. Data are presented as mean ± SD. *p < 0.05, **p < 0.01, ***p < 0.001 versus control

To further explore the inhibitory capability of CY12-RP2 in vivo, A375 cells were injected into the subcutaneous tissue of female BALB/c nude mice to establish a melanoma cells xenograft model, and following a 5 days growth period the tumor-inoculated mice were treated with CY12-RP2 (25 mg/kg/day, 100 mg/kg/day) every two days for 15 days. During this period, the volume of the tumors and the weight of mice were measured every two days. The results showed that CY12-RP2 significantly inhibited the tumor growth when compared with the control group, showing convincing inhibitory rates especially in the high dosage (100 mg/kg) group (Figs. 5A-C). Importantly, there was no significant change in the mean body weight of nude mice (Fig. 5D). Furthermore, the ELISA assay was performed to test the toxicity of CY12-RP2 in hepatic function index, including AST, ALT, TG, and γ-GT. As shown in Fig. S5E, compared with the control group, TG and γ-GT were increased, while AST and ALT have not significantly changed. The HE staining also confirmed that there was no significantly change in the liver, heart, spleen, lung and kidney after CY12-RP2 treatment (Fig. S5F). These results indicated that CY12-RP2 had a slightly toxic effect on the organs of nude mice. Additional TUNEL immunofluorescence assay showed that more apoptotic tumor cells were detected in CY12-RP2-treated group (Fig. S5A), and the level of Ki67 was significantly decreased by CY12-RP2 (Fig. S5B). These results suggested that CY12-RP2 could significantly inhibit the melanoma growth in vivo with slight toxicity. In addition, the in vivo biodistribution of CY12-RP2 was assayed by ELISA. The results suggested that serum levels of CY12-RP2 peaked at 6 h, and moderately decreased progressively with time (Fig. S5C). Organ distribution results showed that CY12-RP2 has a significant ability to target tumors compared to other organs (Fig. S5D).

To assess the efficacy of CY12-RP2 on the growth of allogeneic solid tumors, B16F10-Luc cells were implanted subcutaneously into 6-week-old BALB/c mice. 5 days later, the allograft mouse model was treated with CY12-RP2 (25 mg/kg, 100 mg/kg) every other day through the tail vein for 15 days. The bioluminescence imaging showed significantly decreased tumor growth in the CY12-RP2 high dosage group (100 mg/kg) when compared with the control group (Fig. 5E and F). Moreover, the tumor volume was noticeably decreased by CY12-RP2 (100 mg/kg) (Fig. 5G). Importantly, there was no significant change in the mean body weight of BALB/C mice after CY12-RP2 treatment (Fig. 5H). These results suggested that CY12-RP2 could also inhibit the melanoma cells growth in allogeneic solid tumor model.

### CY12-RP2 enhances the anti-tumor capacity of PBMC in melanoma cells

**Fig. 6:**
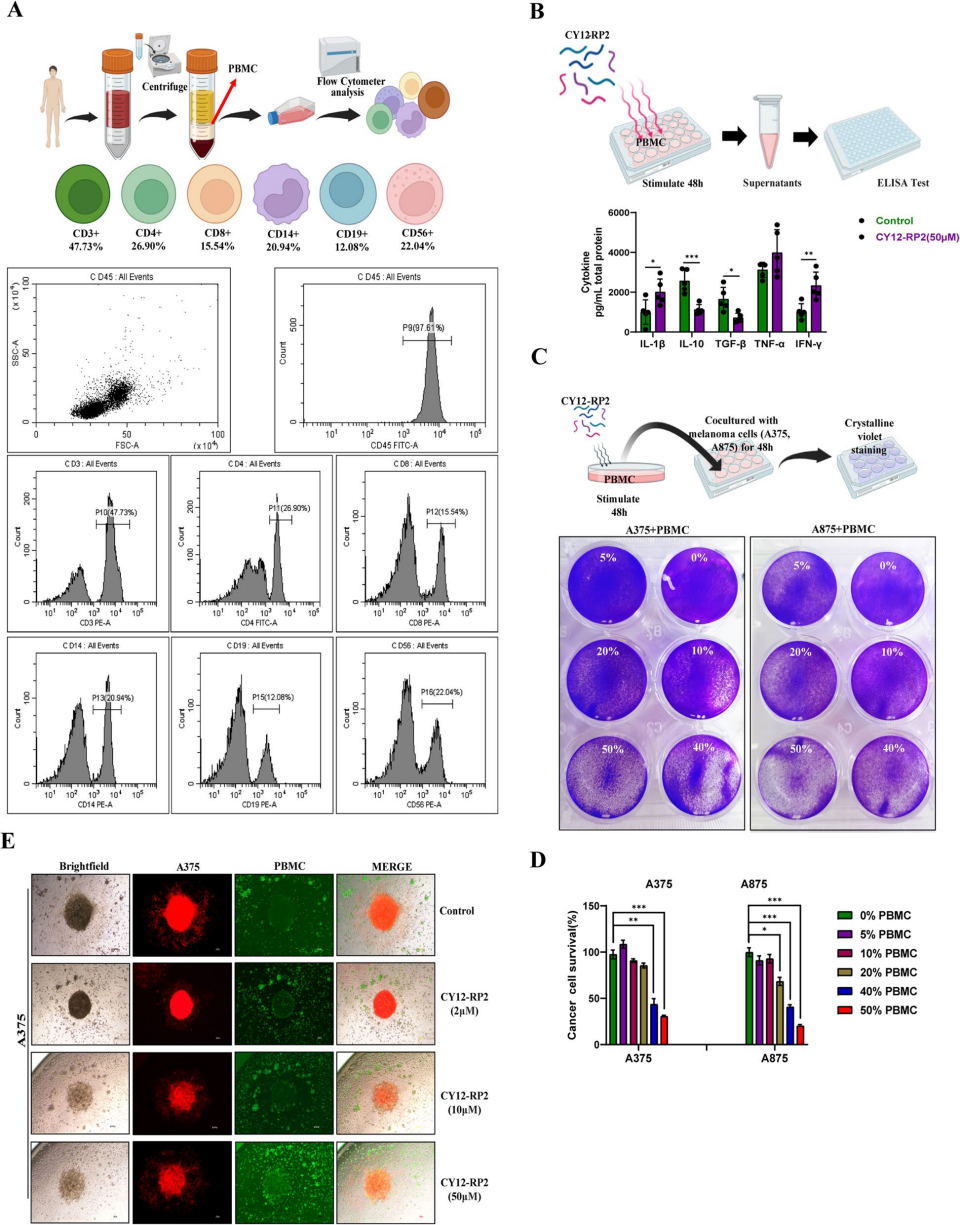
Influence of CY12-RP2 on the immune-mediated anti-tumor capacity of PBMC in vitro and in vivo. **A** The procedure of PBMC isolation from donor peripheral blood, and the ratio of different immune cells in PBMC was assayed by flow cytometry. **B** PBMCs were stimulated with CY12-RP2 for 48 h, and the cytokine levels in the supernatant were assayed by ELISA. **C**, **D** PBMCs stimulated by CY12-RP2 were co-cultured with melanoma cells to demonstrate its killing capacity (**C**), statistical analysis was performed for the killing capacity of PBMC (**D**). **E** Brightfield and fluorescent microscopy images of A375 cells (red) co-culture with PBMC (GFP) were stimulated with various concentrations (0, 2, 10, 50 μM) of CY12-RP2. Data are presented as mean ± SD. *p < 0.05, **p < 0.01, ***p < 0.001 versus control

Chihara et al. have demonstrated that PDPN was a functionally verified immunosuppressive receptor expressed in CD4 + and CD8 + T cells [20]. Previous studies have also demonstrated that PDPN serves as a suppressive element of T cells by restricting the viability and retention of CD4 + effector T cells [20, 39, 40]. These findings strongly suggest that PDPN plays a role in immunosuppression. Therefore, we first investigated the single-cell RNA-sequencing of melanoma immunotherapy from single-cell database (https://singlecell.broadinstitute.org/), and found that PDPN was expressed in multiple immune cells, including T cells, macrophage, NK cells, naive B-cells, etc. (Figs. S6A-C). These results indicated that PDPN may play an important role in tumor immunotherapy. In addition, we investigated the correlation between the expression of PDPN and immune inhibition in SKCM (skin cutaneous melanoma) using the TISIDB database, and found that PDPN was dramatically and positively correlated with immune inhibition (Fig. S6D). To further identify the relationship between PDPN and various immune cells, the correlations between PDPN expression and specific markers of immune cells were investigated through the TIMER database. As shown in Fig. S6E, in skin cutaneous melanoma (SKCM), the PDPN expression was negatively correlated with infiltration levels of various immune cells, including CD4 + T cells, CD8 + T cells, M1 macrophage, and NK cells, and positive correlated with M2 macrophage and Treg cells. These results suggested that high expression of PDPN served an important role in general T cells, macrophages, tumor-associated macrophages, NK cells and Treg cells. The peripheral blood mononuclear cell (PBMC) is defined as any peripheral blood cell, including monocytes and lymphocytes (T cells, B cells, and NK cells). The proportion of immune cells in PBMC was firstly analyzed by flow cytometry, and the results showed that the proportions of CD3 + , CD4 + , CD8 + , CD14 + , CD19 + , and CD56 + cells were 47.3%, 26.90%, 15.54%, 20.94%, 12.08%, and 22.04%, respectively (Fig. 6A). Next, we intended to investigate whether CY12-RP2 could modify PBMC cytokine production. The ELISA results demonstrated that after stimulating PBMC with CY12-RP2 for 48 h, the levels of IL-1β, TNF-α, and IFN-γ in PBMC supernatant increased, while the levels of IL-10 and TGF-β decreased (Fig. 6B). To further confirm whether CY12-RP2 could promote the anti-tumor effect of PBMC, a melanoma cell and PBMC co-culture system was established. The results showed that the viability of tumor cells declined significantly with CY12-RP2-pretreated PBMC in the co-culture system (Fig. 6C and D). Additionally, the 3D melanoma spheroid was used to co-culture with PBMC, and the results showed that with increasing concentrations of CY12-RP2, the tumor-killing capacity of PBMC was increased (Fig. 6E). The above results demonstrated that CY12-RP2 could enhance the anti-tumor effects of PBMC on melanoma cells.

### CY12-RP2 inhibits melanoma lung metastasis in vivo

**Fig. 7:**
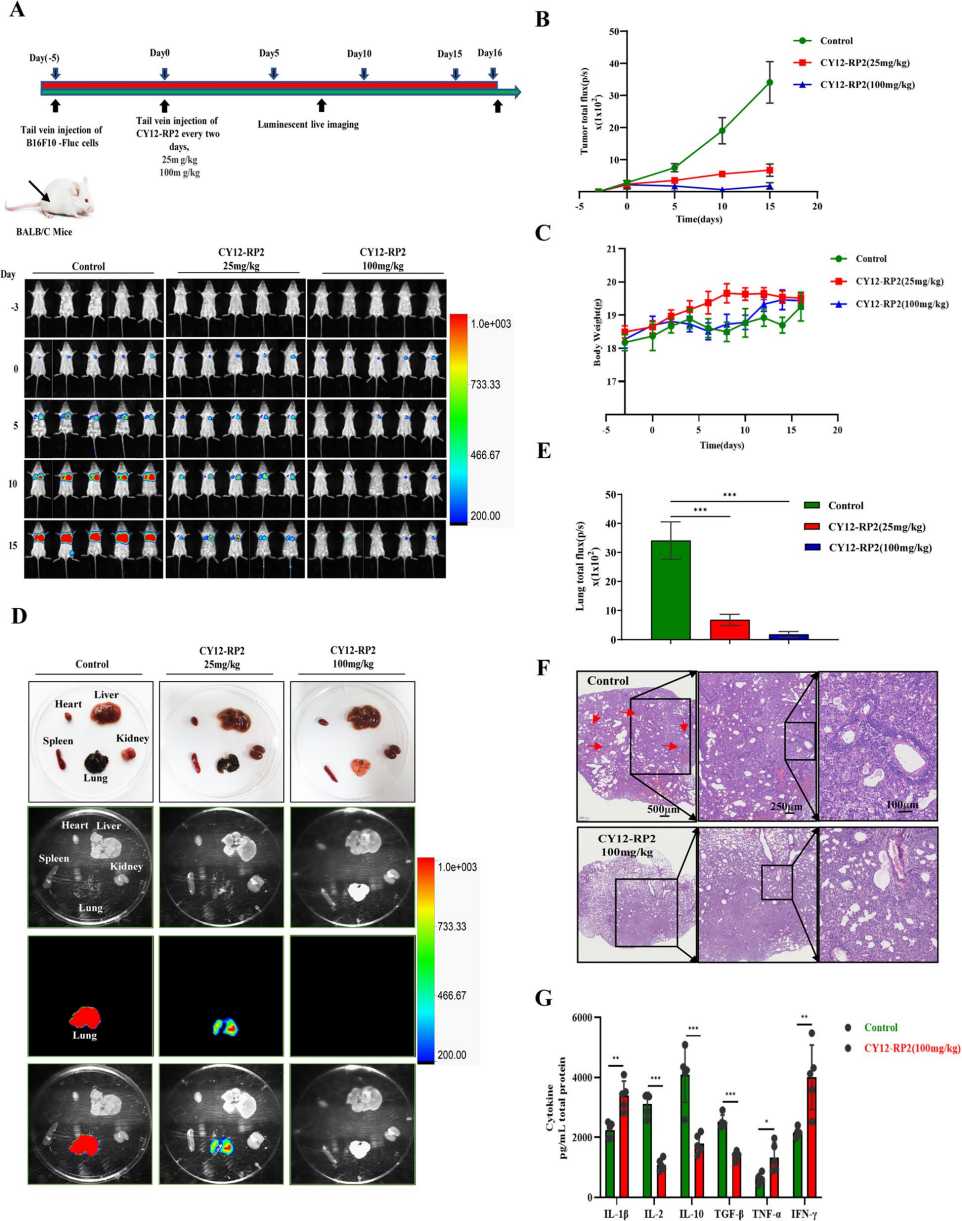
Melanoma cells pulmonary metastasis can be alleviated by CY12-RP2. **A** BALB/c mice were administered intravenously with 5 × 10^5^ melanoma cells B16-F10-Luc and analyzed for lung metastasis for 3 weeks using bioluminescence imaging according to the schematic chart. **B** Statistical analysis of bioluminescence imaging. **C** BALB/c mice's body weight was determined at the indicated time. **D** Organs including heart, liver, spleen, lung, and kidney were imaged using bioluminescence imaging. **E** Quantification analysis of lung metastases fluorescence in BALB/c mice. **F** Representative pulmonary nodules images in a B16-F10 lung metastasis model. The power field scale bar, 500 μm, 250 μm and 100 μm. **G** Cytokine levels including IL-1β, IL-2, IL-10, TGF-β, TNF-α, and IFN-γ in lung metastases model plasma were determined by ELISA. Data are presented as mean ± SD. *p < 0.05, **p < 0.01, ***p < 0.001 versus control

To determine the role of CY12-RP2 on melanoma lung metastasis, we established a lung metastasis model using B16F10-Luc melanoma cells. We found that CY12-RP2 administered BALB/c mice showed significantly fewer pulmonary metastases when compared to the control group (Fig. 7A and B). Corresponding to these discoveries, bioluminescence imaging of the heart, liver, spleen, lung, and kidney displayed a significant reduction in the size of nodules and the frequency of metastases in the lungs with CY12-RP2 treatment (Fig. 7D and E). Furthermore, metastatic nodules in the lung of BALB/c mice were significantly reduced following CY12-RP2 treatment (Fig. 7F). In addition, we observed that there was no significant change in body weight between the treated mice and the control mice (Fig. 7C). Next, we attempted to identify whether CY12-RP2 could modify the levels of immune-related cytokines in mouse plasma by ELISA assays. Cytokine levels of IL-1β, TNF-α, and IFN-γ in the plasma of CY12-RP2 treated lung metastases model were significantly elevated, while the levels of IL-2, IL-10, and TGF-β were reduced when compared with control mice (Fig. 7G). These findings suggested that CY12-RP2 may inhibit melanoma pulmonary metastasis by modulating the secretion of cytokines.

### CY12-RP2 inhibits the melanoma lung metastases through regulating the proportion of immune cells

**Fig. 8:**
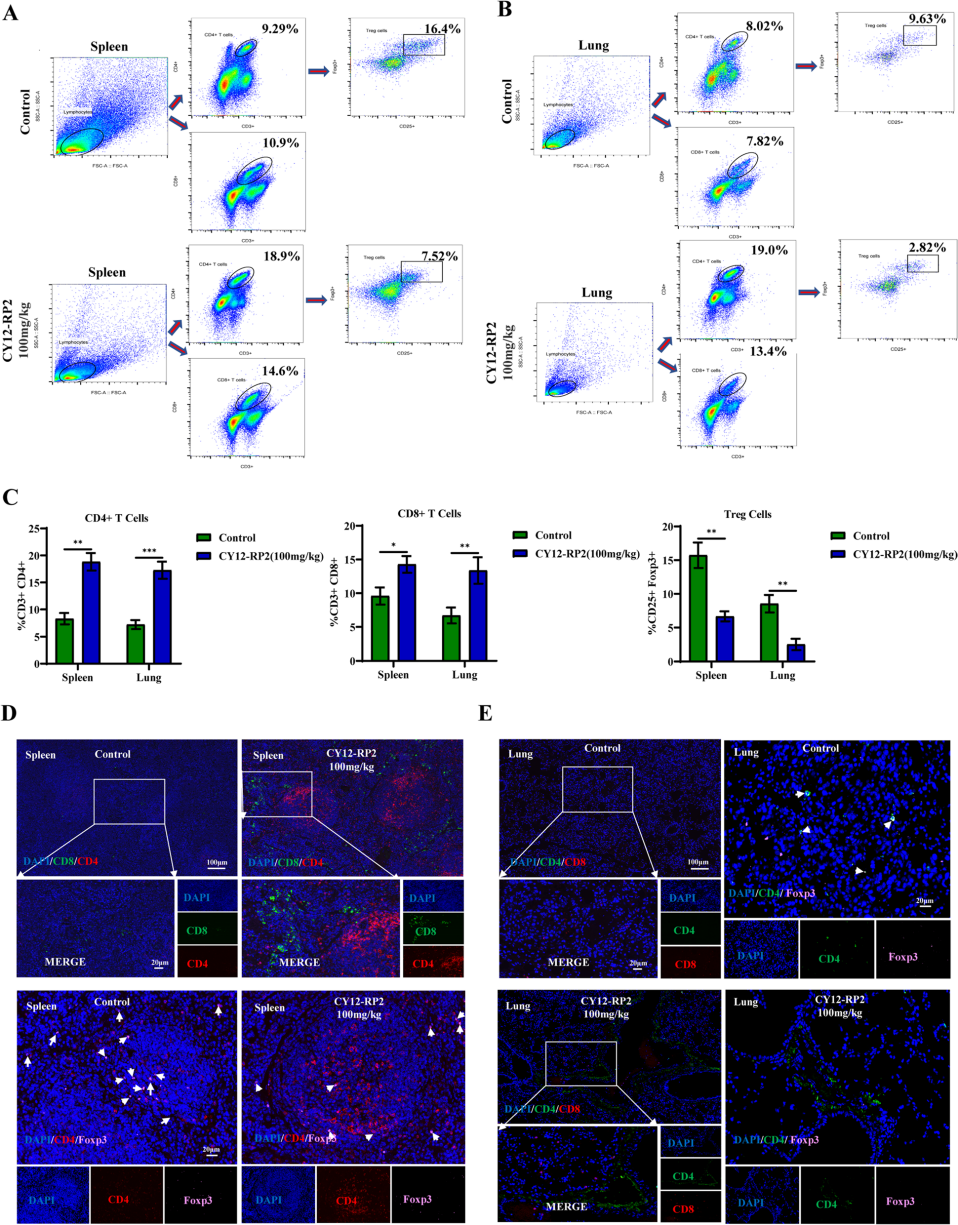
CY12-RP2 modifies the proportion of T cells in the spleen and lung of mice. **A**, **B** The percentage of CD4 + T cells, CD8 + T cells, and CD4 + CD25 + Foxp3 + Treg cells in the spleen and lung of BALB/c mice were detected by flow cytometry. **C** Statistical analysis was performed to count the percentage of CD4 + T cells, CD8 + T cells, and CD4 + CD25 + Foxp3 + Treg cells in the spleen and lung of BALB/c mice with lung metastases. **D**, **E** Representative triple immunofluorescence of T cells (CD4 + T cells, CD8 + T cells) and CD4 + Foxp3 + Treg cells in the spleen (**D**) and lung (**E**) of pulmonary metastasis model. The power field scale bar, 20 μm and 100 μm

In order to assess whether CY12-RP2 could affect the infiltration of immune cells in the spleen and lung of lung metastases mice model, flow cytometry was performed to identify different immune cell populations. We found that the ratios of CD3 + CD4 + T cells and CD3 + CD8 + T cells to total T cells were significantly increased in the lungs and spleens of CY12-RP2-treated metastasis-bearing mice. In contrast, CD4 + CD25 + Foxp3 + Treg cells were significantly diminished (Fig. 8A-C). Furthermore, the proportions of CD49b + Granzyme B + NK cells and CD11b + CD86 + M1-like macrophages were all significantly increased, while the proportion of CD11b + CD206 + M2-like macrophages was decreased in the lungs and spleens after treatment with CY12-RP2 (Figs. S7A, B and S8A, B). The above results were further confirmed by multiplex immunofluorescence analysis, which showed a remarkable increase in infiltration of CD3 + CD4 + T cells and CD3 + CD8 + T cells in the lungs and spleens of CY12-RP2-treated metastasis-bearing mice, together with the reduction in infiltration of Treg cells (Fig. 8D and E). In addition, we also examined the immune infiltration of NK cells and macrophages in the lungs and spleens by immunofluorescence. The results showed that CY12-RP2-treated mice showed increased proportions of NK cells and M1-type macrophages and a decreased proportion of M2-type macrophages in their lungs and spleens (Figs. S7C and S8C, D). Thus, our results collectively indicated that CY12-RP2 could inhibit the progression of melanoma lung metastasis through mainly modulating the proportions of lymphocytes, NK cells, and macrophages.

## Discussion

Melanoma is potentially the most aggressive and fatal type of skin cancers [2]. Current therapeutic options for melanoma are varied [5, 6], with the treatment regimen determined by the patient's tumor stage and location, and may either include single agent or combination therapy. Targeted therapy is an approach to cancer treatment that utilizes therapeutics that can precisely target and attack cancer cells [8]. The development of drugs that target constitutively and specifically expressed proteins on the surface of melanoma cells plays an integral role in melanoma therapy. Various studies have shown that PDPN expression is significantly elevated in melanoma clinical tissue and cells, and is frequently associated with poor prognosis [15]. PDPN is also increasingly acknowledged as a critical cellular protein associated with an assortment of functions, including cell motility, metastasis, lymphangiogenesis, organogenesis, platelet production, and tumorigenesis as well as promotion of inflammatory disease and immunoevasion [15, 41]. We herein sought to gain a better insight into the function of PDPN and the potential of an antagonist in melanoma treatment. Firstly, we have demonstrated that PDPN expression was closely associated with melanoma development. Furthermore, we used Ph.D.TM-12 Phage Display Peptide Library to obtain the PDPN antagonist peptide CY12-RP2, and found that CY12-RP2 could significantly inhibit the melanoma growth and metastasis both in vitro and in vivo.

The dysregulation of the Wnt/β-catenin signaling pathway is frequently associated with the development of melanoma [42]. Specifically, Wnt/β-catenin signaling pathway related genes such as β-catenin, GSK3β, Anxin, and DVL are pivotal to melanocyte development and pathogenesis of melanoma, directing neural crest cell differentiation to form melanocytes and driving melanoma cell proliferation and invasion [43, 44]. Jiaqi et al. found that Wnt/β-catenin signalosome transduction may be regulated by the various functional regions of PDPN, including the transmembrane and cytoplasmic domain [45]. In addition, the transmembrane domain of PDPN associates with CD44 to facilitate directional cell migration may require activation of Wnt signaling pathway [21]. In our study, we found that PDPN was overexpressed in melanoma clinical tissue and cells, and PDPN could promote melanoma cell migration and invasion through activating the Wnt/β-catenin pathway. Treatment of melanoma cells with shRNA lentivirus to knockdown PDPN or with PDPN antagonist peptide CY12-RP2 could inhibit the Wnt/β-catenin pathway, which suggested that PDPN promoted melanoma cell proliferation, migration, and invasion at least partially via activation of the Wnt/β-catenin signaling pathway. Besides its effects on melanoma cells, the Wnt/β-catenin signaling pathway also influences the immune response, but the roles of Wnt/β-catenin in the melanoma immune system have not yet been well characterized. Intriguingly, recent findings have indicated that β-catenin was involved in immune suppression and tolerance. Overexpression of β-catenin in Tregs has been reported to significantly increase the survival of those cells [46, 47]. Furthermore, Luke et al. assessed 8890 tumor samples and classified them into T cell-inflamed, non-T cell inflamed, and intermediate subtypes from TCGA database. They further found that Wnt/β-catenin signaling pathway activation and mutations were associated with T-cell inflammatory gene by exon sequencing. Immunohistochemistry of tumor tissues showed that β-catenin was inversely correlated with CD8 + T cell infiltration [48]. Consistent with our results, active Wnt/β-catenin signaling was inversely correlated with T-cell infiltration in melanoma mouse models. Thus, targeting the Wnt/β-catenin signaling pathway in immune cells (e.g., T lymphocyte, NK cells and macrophage) may restore the patient's immunocompetence. Historical studies have shown that melanoma is also one of the most immunomodulatory-sensitive malignancies, and intensive infiltration being correlated to favorable prognosis [49, 50]. We speculate that there may be interactions between PDPN and tumor-infiltrating immune cells. The results derived from database analysis demonstrated that PDPN displayed a significant effect on immune infiltration or polarization because of its positive association with increased infiltration levels of various immune cells (Figure S6A and E). We speculated this may be due to CLEC-2 being predominantly expressed on platelets and bone marrow immune cells like T cells, macrophages, and NK cells. Moreover, PDPN is expressed on hematopoietic cells, including subpopulations of T cells and macrophages, which is beneficial for CLEC-2 binding with PDPN to promote melanoma growth and metastasis through regulating immune cells.

The tumor-infiltrating lymphocytes (TILs) are closely correlated with tumor checkpoint blockade immunotherapy [51]. In solid tumor microenvironment, various regulatory pathways regulate the chemokines, immunomodulatory enzymes, T regulatory cells, and macrophages to impair the accumulation and function of TILs [52]. Hatzioannou et al. found that PDPN-expressing lymph node stromal cells (LNSCs) could promote melanoma growth through elimination of TILs. Blocking the PDPN + LNSCs by LTbR-Ig could increase the infiltration and proliferation of CD4 + TILs and inhibit melanoma development [53]. Development of PDPN targeted therapeutics may be a useful method to enhance the effects of immunotherapy in melanoma. To this end, the PDPN antagonist peptide, CY12-RP2, was obtained and validated to bind specifically with PDPN. In vitro studies utilizing CY12-RP2 resulted in a decrease in cell proliferation, migration, invasion of melanoma cells, and an increase the anti-tumor capacity of PBMC in melanoma cells. For the in vivo studies, we found that CY12-RP2 could not only inhibit the melanoma growth, but also suppress the melanoma lung metastasis. Moreover, both the FACS and immunofluorescence assays showed that CY12-RP2 targeting PDPN was positively correlated with T cell (CD4 + , CD8 + T cells) infiltration and negatively correlated with Treg cells infiltration (Fig. 8A-E). These results imply that CY12-RP2 may exercise its anti-tumor effects through increasing the infiltration and proliferation of TILs.

Macrophages are the main type of immune cells in the tumor microenvironment, which can be divided into two types: M1 and M2 macrophages [54]. The M1 macrophage exert a proinflammatory effect and secrete cytokines, like IL-6 and IL-12, to inhibit tumor growth through phagocytosis, proinflammatory response, and activation of immune response [54]. M2 macrophages are characterized by markers such as CD206 + , CD163 + , CD86-, and can induce the suppression of the immune response through production of IL-10 and TGF-β [55]. Tumor-associated macrophages (TAMs) are closely correlated with M2 polarization, which can promote tumor growth and metastasis through release of platelet-derived growth factor and vascular endothelial growth factor [56]. The Wnt/β-catenin pathway is important for monocyte-to-macrophage differentiation and macrophages M2 polarization. Yang et al. found that there is abundant nuclear β-catenin accumulation in M2 CD68 + macrophages from human HCC biopsies, and decreasing the level of β-catenin in M2 macrophages could inhibit HCC growth [57]. Therefore, targeting TAMs to reprogram M2 macrophage to repolarize into M1 is a promising useful method for tumor immunotherapy. In our study, the peptide CY12-RP2 could block the Wnt/β-catenin signaling pathway, increase the proportion of CD11b + CD86 + M1-like macrophages, and decrease the proportion of CD11b + CD206 + M2-like macrophages and the level of IL-2, IL-10, and TGF-β. These results imply that CY12-RP2 could inhibit the melanoma growth and metastasis through reprograming the TME. CY12-RP2 may be developed into a therapeutic to improve the efficacy of immunotherapy for treating melanoma.

## Conclusions

Taken altogether, our study investigated the role of PDPN in melanoma, and confirmed that PDPN could promote melanoma growth and metastasis through activating the Wnt/β-catenin pathway. We then developed a novel PDPN antagonistic peptide drug, CY12-RP2, through phage display technology. CY12-RP2 could inhibit melanoma cell growth and metastasis in vivo and in vitro through Wnt/β-catenin pathway blockade. In addition, CY12-RP2 could abrogate the immunosuppressive effects of PDPN on immune cells. These results elucidate an important role of PDPN in maintaining melanoma growth and progression, and provide a novel therapeutic peptide CY12-RP2 for melanoma treatment.

### Supplementary Information

**Additional file 1: Fig. S1.** PDPN was upregulated in melanoma, which was prognostically relevant to melanoma patient survival. **Fig. S2.** The effects of PDPN on migration and invasion could be modulated by SKL2001and IWR1. **Fig. S3.** The identification and functional analysis of PDPN- binding peptides. **Fig. S4.** Effects and toxicity of CY12-RP2 in vivo. **Fig. S5.** The inhibition of cells metastasis and invasion by CY12-RP2 can be reversed by SKL2001. **Fig. S6.** The expression of PDPN is associated with immune infiltration of melanoma tumors. **Fig. S7.** CY12-RP2 modifies the population of NK cells in the spleen and lung of mice. **Fig. S8.** The effects of CY12-RP2 on the proportion of macrophages (M1 and M2) in the spleen and lung of mice. **Table 1.** Antibodies applied for western-blotting, flow cytometry, immunofluorescence and immunohistochemistry analysis

## Data Availability

All the data are present in the manuscript or in the Supplementary figures.

## Abbreviations

PDPN: Podoplanin
EMT: Epithelial-mesenchymal transition
TCGA: The Cancer Genome Atlas
PBMC: Peripheral Blood Mononuclear Cell
Treg: Regulatory T cells
IFN-γ: Interferon-γ
IL-1β: Interleukin-1β
TNF-α: Tumor necrosis factor α
TGF-β: Transforming growth factor-β
CLEC-2: C-type lectin receptor-2
TAM: Tumor-associated macrophages
RT-PCR: Real-time quantitative PCR
ELISA: Enzyme-linked immunosorbent assay

## References

[1] Gupta AK, Bharadwaj M, Mehrotra R (2016). Skin cancer concerns in people of color: risk factors and prevention. Asian Pac J Cancer Prev.

[2] van der Weyden L, Brenn T, Patton EE, Wood GA, Adams DJ (2020). Spontaneously occurring melanoma in animals and their relevance to human melanoma. J Pathol.

[3] Domingues B, Lopes JM, Soares P, Populo H (2018). Melanoma treatment in review. Immunotargets Ther.

[4] Bertrand JU, Steingrimsson E, Jouenne F, Bressac-de Paillerets B, Larue L (2020). Melanoma Risk and Melanocyte Biology. Acta Derm Venereol.

[5] Tyrell R, Antia C, Stanley S, Deutsch GB (2017). Surgical resection of metastatic melanoma in the era of immunotherapy and targeted therapy. Melanoma Manag.

[6] Switzer B, Puzanov I, Skitzki JJ, Hamad L, Ernstoff MS (2020). Managing metastatic melanoma in 2022: a clinical review. JCO Oncol Pract.

[7] Menon D, Das S, Rinner B, Heike K, Bonyadirad E, Hoefler G, Schaider H. Transient CD271+ve Drug Tolerant Stem Like Melanoma Cells Are Inherently Resistant Towards BRAF And MEK Inhibition. J Investig Dermatol. 2012;132:S124.

[8] Subbiah V, Baik C, Kirkwood JM (2020). Clinical development of BRAF plus MEK Inhibitor Combinations. Trends Cancer.

[9] Sikorska J, Gawel D, Domek H, Rudzinska M, Czarnocka B (2019). Podoplanin (PDPN) affects the invasiveness of thyroid carcinoma cells by inducing ezrin, radixin and moesin (E/R/M) phosphorylation in association with matrix metalloproteinases. BMC Cancer.

[10] Krishnan H, Rayes J, Miyashita T, Ishii G, Retzbach EP, Sheehan SA, Takemoto A, Chang YW, Yoneda K, Asai J, Jensen L, Chalise L, Natsume A, Goldberg GS (2018). Podoplanin: an emerging cancer biomarker and therapeutic target. Cancer Sci.

[11] Suzuki H, Kaneko MK, Kato Y (2022). Roles of Podoplanin in Malignant Progression of Tumor. Cells.

[12] Shinada M, Kato D, Kamoto S, Yoshimoto S, Tsuboi M, Yoshitake R, Eto S, Ikeda N, Saeki K, Hashimoto Y, Takahashi Y, Chambers J, Uchida K, Kaneko MK, Fujita N, Nishimura R, Kato Y, Nakagawa T (2020). PDPN Is Expressed in various types of canine tumors and its silencing induces apoptosis and cell cycle arrest in canine Malignant Melanoma. Cells.

[13] Astarita JL, Acton SE, Turley SJ (2012). Podoplanin: emerging functions in development, the immune system, and cancer. Front Immunol.

[14] Kabashima-Niibe A, Higuchi H, Takaishi H, Masugi Y, Matsuzaki Y, Mabuchi Y, Funakoshi S, Adachi M, Hamamoto Y, Kawachi S, Aiura K, Kitagawa Y, Sakamoto M, Hibi T (2013). Mesenchymal stem cells regulate epithelial-mesenchymal transition and tumor progression of pancreatic cancer cells. Cancer Sci.

[15] Asai J (2022). The role of podoplanin in skin diseases. Int J Mol Sci.

[16] Martin-Villar E, Fernandez-Munoz B, Parsons M, Yurrita MM, Megias D, Perez-Gomez E, Jones GE, Quintanilla M (2010). Podoplanin associates with CD44 to promote directional cell migration. Mol Biol Cell.

[17] Bresson L, Faraldo MM, Di-Cicco A, Quintanilla M, Glukhova MA, Deugnier MA (2018). Podoplanin regulates mammary stem cell function and tumorigenesis by potentiating Wnt/β-catenin signaling. Development.

[18] Kovacs D, Migliano E, Muscardin L, Silipo V, Catricalà C, Picardo M, Bellei B (2016). The role of WNT/β-catenin signaling pathway in melanoma epithelial-to-mesenchymal-like switching: evidences from patients-derived cell lines. Oncotarget.

[19] Sesartić M (2020). The role of keratinocyte-expressed podoplanin in skin carcinogenesis and wound healing.

[20] Suzuki-Inoue K, Osada M, Ozaki Y (2017). Physiologic and pathophysiologic roles of interaction between C-type lectin-like receptor 2 and podoplanin: partners from in utero to adulthood. J Thromb Haemost.

[21] Ochoa-Alvarez JA, Krishnan H, Shen Y, Acharya NK, Han M, McNulty DE, Hasegawa H, Hyodo T, Senga T, Geng JG, Kosciuk M, Shin SS, Goydos JS, Temiakov D, Nagele RG, Goldberg GS (2012). Plant lectin can target receptors containing sialic acid, exemplified by podoplanin, to inhibit transformed cell growth and migration. PLoS ONE.

[22] Izci M, Maksoudian C, Manshian BB, Soenen SJ (2021). The use of alternative strategies for enhanced nanoparticle delivery to solid Tumors. Chem Rev.

[23] Bambace NM, Holmes CE (2011). The platelet contribution to cancer progression. J Thromb Haemost.

[24] Obermann WMJ, Brockhaus K, Eble JA (2021). Platelets, constant and cooperative companions of sessile and disseminating tumor cells, crucially contribute to the tumor microenvironment. Front Cell Dev Biol.

[25] Braun A, Anders HJ, Gudermann T, Mammadova-Bach E (2021). Platelet-cancer interplay: molecular mechanisms and new therapeutic avenues. Front Oncol.

[26] Liu J, Xiao Q, Xiao J, Niu C, Li Y, Zhang X, Zhou Z, Shu G, Yin G (2022). Wnt/beta-catenin signalling: function, biological mechanisms, and therapeutic opportunities. Signal Transduct Target Ther.

[27] Hu L, Zhang P, Sun W, Zhou L, Chu Q, Chen Y (2020). PDPN is a prognostic biomarker and correlated with immune infiltrating in gastric cancer. Medicine (Baltimore).

[28] Chiangjong W, Chutipongtanate S, Hongeng S (2020). Anticancer peptide: Physicochemical property, functional aspect and trend in clinical application (Review). Int J Oncol.

[29] Jaroszewicz W, Morcinek-Orlowska J, Pierzynowska K, Gaffke L, Wegrzyn G (2022). Phage display and other peptide display technologies. FEMS Microbiol Rev.

[30] Karami Fath M, Babakhaniyan K, Zokaei M, Yaghoubian A, Akbari S, Khorsandi M, Soofi A, Nabi-Afjadi M, Zalpoor H, Jalalifar F, Azargoonjahromi A, Payandeh Z, Alagheband Bahrami A (2022). Anti-cancer peptide-based therapeutic strategies in solid tumors. Cell Mol Biol Lett.

[31] Zhang G, Li C, Quartararo AJ, Loas A, Pentelute BL (2021). Automated affinity selection for rapid discovery of peptide binders. Chem Sci.

[32] Saw PE, Song EW (2019). Phage display screening of therapeutic peptide for cancer targeting and therapy. Protein Cell.

[33] Araste F, Abnous K, Hashemi M, Taghdisi SM, Ramezani M, Alibolandi M (2018). Peptide-based targeted therapeutics: Focus on cancer treatment. J Control Release.

[34] Matsuo AL, Tanaka AS, Juliano MA, Rodrigues EG, Travassos LR (2010). A novel melanoma-targeting peptide screened by phage display exhibits antitumor activity. J Mol Med (Berl).

[35] Deng T, Hou Y, Lin G, Feng C, Liu K, Chen W, Wei W, Huang L, Dai X (2023). A Novel Fibromodulin antagonist peptide RP4 Exerts Antitumor Effects on Colorectal Cancer. Pharmaceutics.

[36] Wang K, Dai X, Yu A, Feng C, Liu K, Huang L (2022). Peptide-based PROTAC degrader of FOXM1 suppresses cancer and decreases GLUT1 and PD-L1 expression. J Exp Clin Cancer Res.

[37] Yang S, Sun S, Xu W, Yu B, Wang G, Wang H (2020). Astragalus polysaccharide inhibits breast cancer cell migration and invasion by regulating epithelial-mesenchymal transition via the Wnt/beta-catenin signaling pathway. Mol Med Rep.

[38] Zhang Y, Wang X (2020). Targeting the Wnt/beta-catenin signaling pathway in cancer. J Hematol Oncol.

[39] Mao X, Xu J, Wang W, Liang C, Hua J, Liu J, Zhang B, Meng Q, Yu X, Shi S (2021). Crosstalk between cancer-associated fibroblasts and immune cells in the tumor microenvironment: new findings and future perspectives. Mol Cancer.

[40] Liu X, Cao Y, Lv K, Gu Y, Jin K, He X, Fang H, Fei Y, Shi M, Lin C, Liu H, Li H, He H, Xu J, Li R, Zhang H (2020). Tumor-infiltrating podoplanin(+) cells in gastric cancer: clinical outcomes and association with immune contexture. Oncoimmunology.

[41] Watanabe N, Kidokoro M, Tanaka M, Inoue S, Tsuji T, Akatuska H, Okada C, Iida Y, Okada Y, Suzuki Y, Sato T, Yahata T, Hirayama N, Nakagawa Y, Inokuchi S (2020). Podoplanin is indispensable for cell motility and platelet-induced epithelial-to-mesenchymal transition-related gene expression in esophagus squamous carcinoma TE11A cells. Cancer Cell Int.

[42] Gajos-Michniewicz A, Czyz M (2020). WNT Signaling in Melanoma. Int J Mol Sci.

[43] Katkat E, Demirci Y, Heger G, Karagulle D, Papatheodorou I, Brazma A, Ozhan G. Canonical Wnt and TGF-β/BMP signaling enhance melanocyte regeneration and suppress invasiveness, migration, and proliferation of melanoma cells. bioRxiv. 2022;11:483949. 10.3389/fcell.2023.1297910 PMC10679360 38020918

[44] Pond KW, Doubrovinski K, Thorne CA (2020). Wnt/beta-catenin Signaling in Tissue Self-Organization. Genes (Basel).

[45] de Winde CM, Makris S, Millward L, Rebordinos JC, Benjamin AC, Martínez VG, Acton SE (2019). Podoplanin function is switched by partner proteins on fibroblastic reticular cells.

[46] Ding Y, Shen S, Lino AC (2008). Beta-catenin stabilization extends regulatory T cell survival and induces anergy in nonregulatory T cells. Nat Med.

[47] Manicassamy S, Reizis B, Ravindran R (2010). Activation of β-catenin in dendritic cells regulates immunity versus tolerance in the intestine. Science.

[48] Luke JJ, Bao R, Spranger S (2016). Correlation of WNT/β-catenin pathway activation with immune exclusion across most human cancers.

[49] Wang P, Zhang X, Sun N, Zhao Z, He J (2020). Comprehensive Analysis of the Tumor Microenvironment in Cutaneous Melanoma associated with Immune Infiltration. J Cancer.

[50] Caraban BM, Matei E, Cozaru GC, Aschie M, Deacu M, Enciu M, Baltatescu GI, Chisoi A, Dobrin N, Petcu L, Gheorghe E, Hangan LT, Rosu MC, Orasanu CI, Nicolau AA (2023). PD-L1, CD4+, and CD8+ Tumor-Infiltrating Lymphocytes (TILs) Expression Profiles in Melanoma Tumor Microenvironment Cells. J Pers Med.

[51] Gajewski TF, Schreiber H, Fu YX (2013). Innate and adaptive immune cells in the tumor microenvironment. Nat Immunol.

[52] Zou W (2005). Immunosuppressive networks in the tumour environment and their therapeutic relevance. Nat Rev Cancer.

[53] Cueni LN, Hegyi I, Shin JW, Albinger-Hegyi A, Gruber S, Kunstfeld R, Moch H, Detmar M (2010). Tumor lymphangiogenesis and metastasis to lymph nodes induced by cancer cell expression of podoplanin. Am J Pathol.

[54] Xiang X, Wang J, Lu D, Xu X (2021). Targeting tumor-associated macrophages to synergize tumor immunotherapy. Signal Transduct Target Ther.

[55] Pan Y, Yu Y, Wang X, Zhang T (2020). Tumor-Associated Macrophages in Tumor Immunity. Front Immunol.

[56] Han S, Wang W, Wang S, Yang T, Zhang G, Wang D, Ju R, Lu Y, Wang H, Wang L (2021). Tumor microenvironment remodeling and tumor therapy based on M2-like tumor associated macrophage-targeting nano-complexes. Theranostics.

[57] Yang Y, Ye YC, Chen Y, Zhao JL, Gao CC, Han H, Liu WC, Qin HY (2018). Crosstalk between hepatic tumor cells and macrophages via Wnt/β-catenin signaling promotes M2-like macrophage polarization and reinforces tumor malignant behaviors. Cell Death Dis.

