# Supplementary material for: A novel PDPN antagonist peptide CY12-RP2 inhibits melanoma growth via Wnt/β-catenin and modulates the immune cells
Source: J Exp Clin Cancer Res. 2024 Jan 2;43:9. doi: 10.1186/s13046-023-02910-y (PMC10759609; doi:10.1186/s13046-023-02910-y)
Supplement: Supplementary file 1 — Additional file 1: Fig. S1. PDPN was upregulated in melanoma, which was prognostically relevant to melanoma patient survival. Fig. S2. The effects of PDPN on migration and invasion could be modulated by SKL2001and IWR1. Fig. S3. The identification and functional analysis of PDPN- binding peptides. Fig. S4. Effects and toxicity of CY12-RP2 in vivo. Fig. S5. The inhibition of cells metastasis and invasion by CY12-RP2 can be reversed by SKL2001. Fig. S6. The expression of PDPN is associated with immune infiltration of melanoma tumors. Fig. S7. CY12-RP2 modifies the population of NK cells in the spleen and lung of mice. Fig. S8. The effects of CY12-RP2 on the proportion of macrophages (M1 and M2) in the spleen and lung of mice. Table 1. Antibodies applied for western-blotting, flow cytometry, immunofluorescence and immunohistochemistry analysis [file 13046_2023_2910_MOESM1_ESM.pdf]

Supplement

Figures and table included

A Novel PDPN Antagonist Peptide CY12-RP2 inhibits melanoma growth

via Wnt/β-catenin and modulates the immune cells

Fig. S1

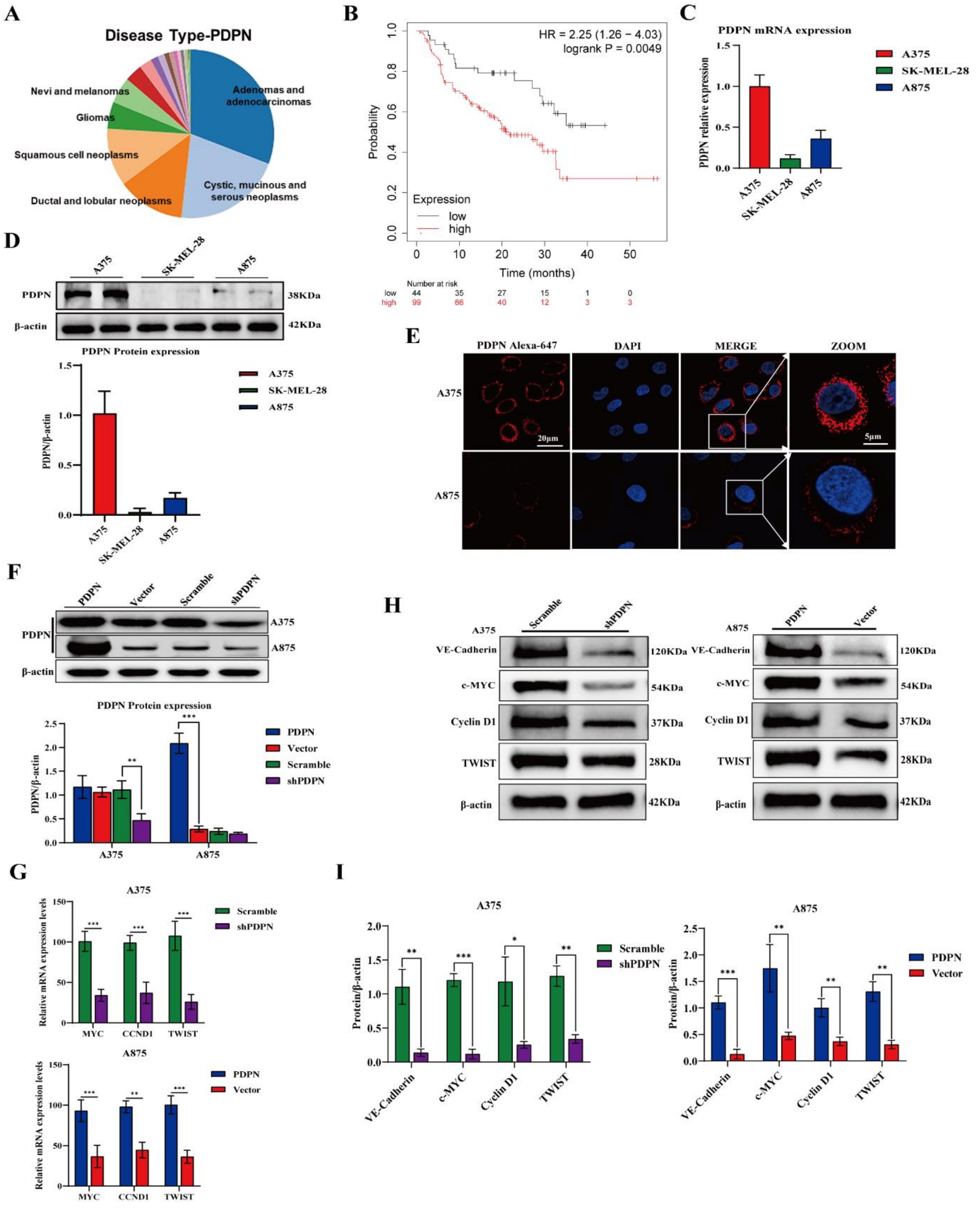
A
Disease Type-PDPN
Adenomas and adenocarcinomas
Cystic, mucinous and serous neoplasms
Ductal and lobular neoplasms
Squamous cell neoplasms
Gliomas
Nevi and melanomas
B
HR = 2.25 (1.26 − 4.03)
logrank P = 0.0049
Probability
Time (months)
Expression
low
high
Number at risk
low 44 35 27 15 1 0
high 99 66 40 12 3 3
C
PDPN mRNA expression
PDPN relative expression
A375
SK-MEL-28
A875
D
PDPN
β-actin
38KDa
42KDa
PDPN Protein expression
PDPN/β-actin
E
PDPN Alexa-647
DAPI
MERGE
ZOOM
20μm
5μm
F
PDPN
Vector
Scramble
shPDPN
PDPN/β-actin
G
Relative mRNA expression levels
MYC
CCND1
TWIST
H
VE-Cadherin
c-MYC
Cyclin D1
TWIST
120KDa
54KDa
37KDa
28KDa
I
Protein/β-actin

Fig. S2

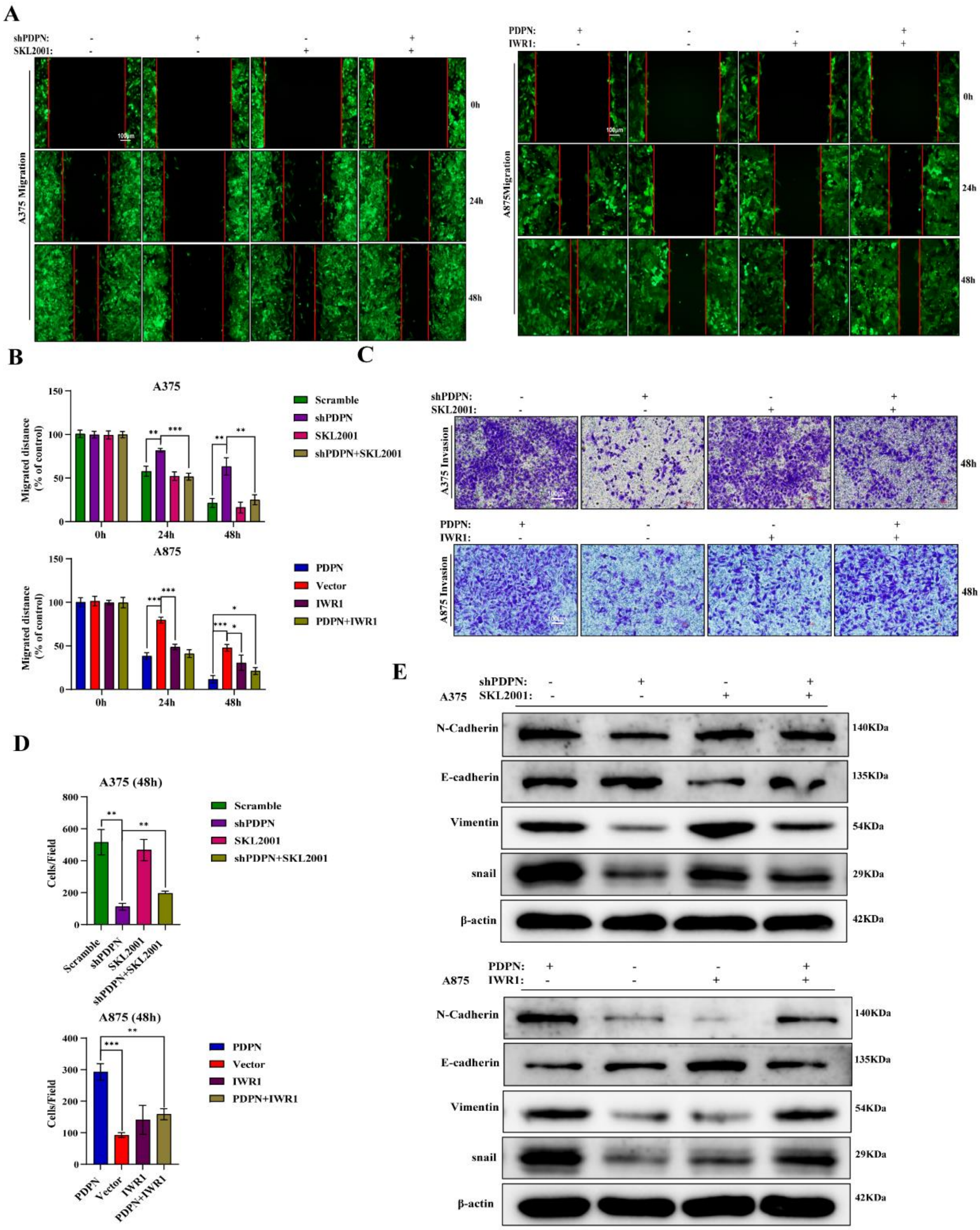

A
shPDPN: - + - +
SKL2001: - - + +
A375 Migration
0h
24h
48h
100μm
PDPN: + - - +
IWR1: - - + +
A875Migration
B
A375
Migrated distance (% of control)
Scramble
shPDPN
SKL2001
shPDPN+SKL2001
A875
PDPN
Vector
IWR1
PDPN+IWR1
C
A375 Invasion
A875 Invasion
D
A375 (48h)
Cells/Field
A875 (48h)
E
N-Cadherin 140KDa
E-cadherin 135KDa
Vimentin 54KDa
snail 29KDa
β-actin 42KDa

Fig. S3

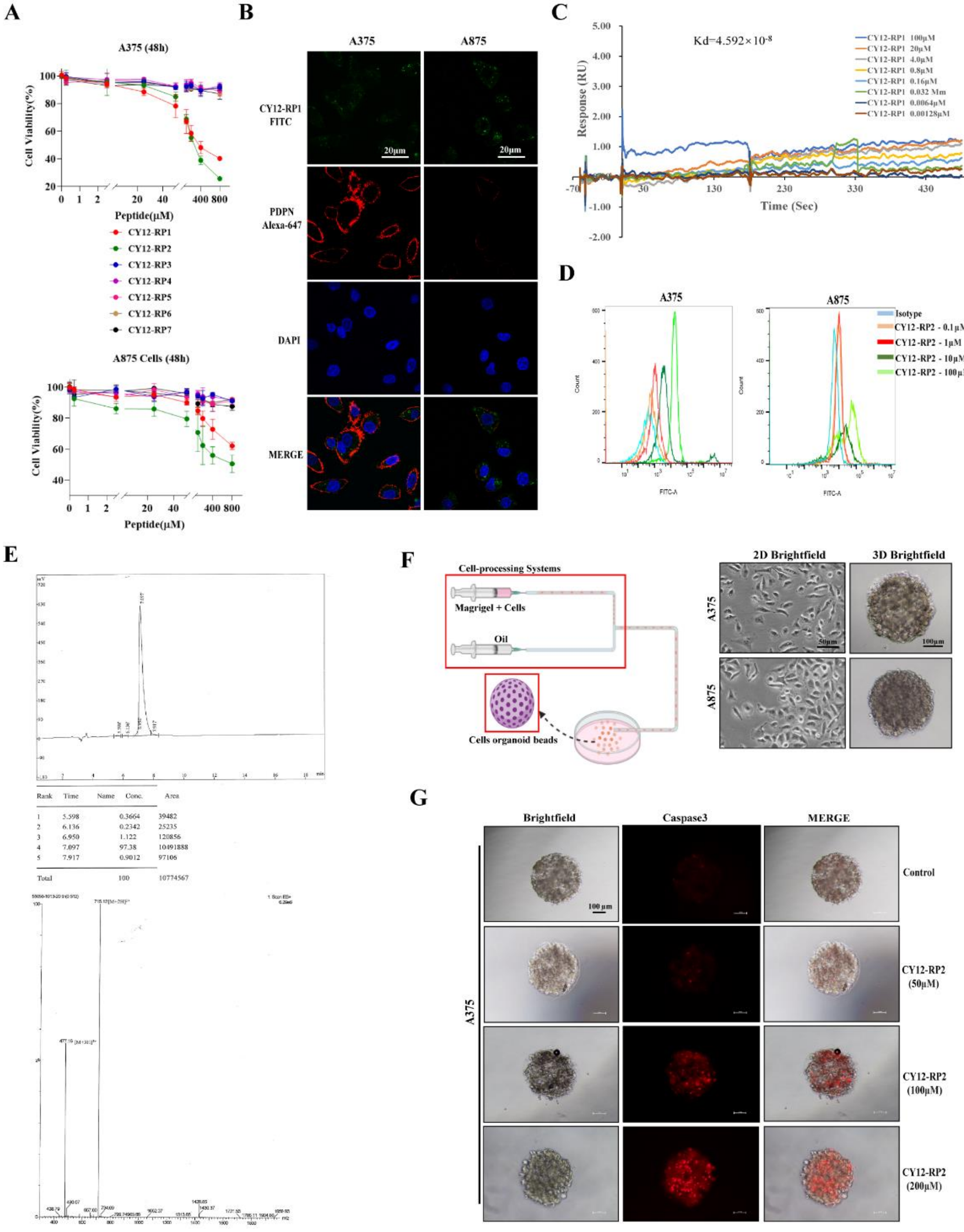
A
A375 (48h)
Cell Viability(%)
Peptide(μM)
CY12-RP1
CY12-RP2
CY12-RP3
CY12-RP4
CY12-RP5
CY12-RP6
CY12-RP7
A875 Cells (48h)
B
A375
A875
CY12-RP1 FITC
PDPN Alexa-647
DAPI
MERGE
20μm
C
Kd=4.592×10-8
Response (RU)
Time (Sec)
CY12-RP1 100μM
CY12-RP1 20μM
CY12-RP1 4.0μM
CY12-RP1 0.8μM
CY12-RP1 0.16μM
CY12-RP1 0.032 Mm
CY12-RP1 0.0064μM
CY12-RP1 0.00128μM
D
Isotype
CY12-RP2 - 0.1μM
CY12-RP2 - 1μM
CY12-RP2 - 10μM
CY12-RP2 - 100μM
FITC-A
Count
E
Rank Time Name Conc. Area
1 5.598 0.3664 39482
2 6.136 0.2342 25235
3 6.950 1.122 120856
4 7.097 97.38 10491888
5 7.917 0.9012 97106
Total 100 10774567
F
Cell-processing Systems
Magrigel + Cells
Oil
Cells organoid beads
2D Brightfield
3D Brightfield
50μm
100μm
G
Brightfield
Caspase3
MERGE
Control
CY12-RP2 (50μM)
CY12-RP2 (100μM)
CY12-RP2 (200μM)
100 μm

Fig. S4

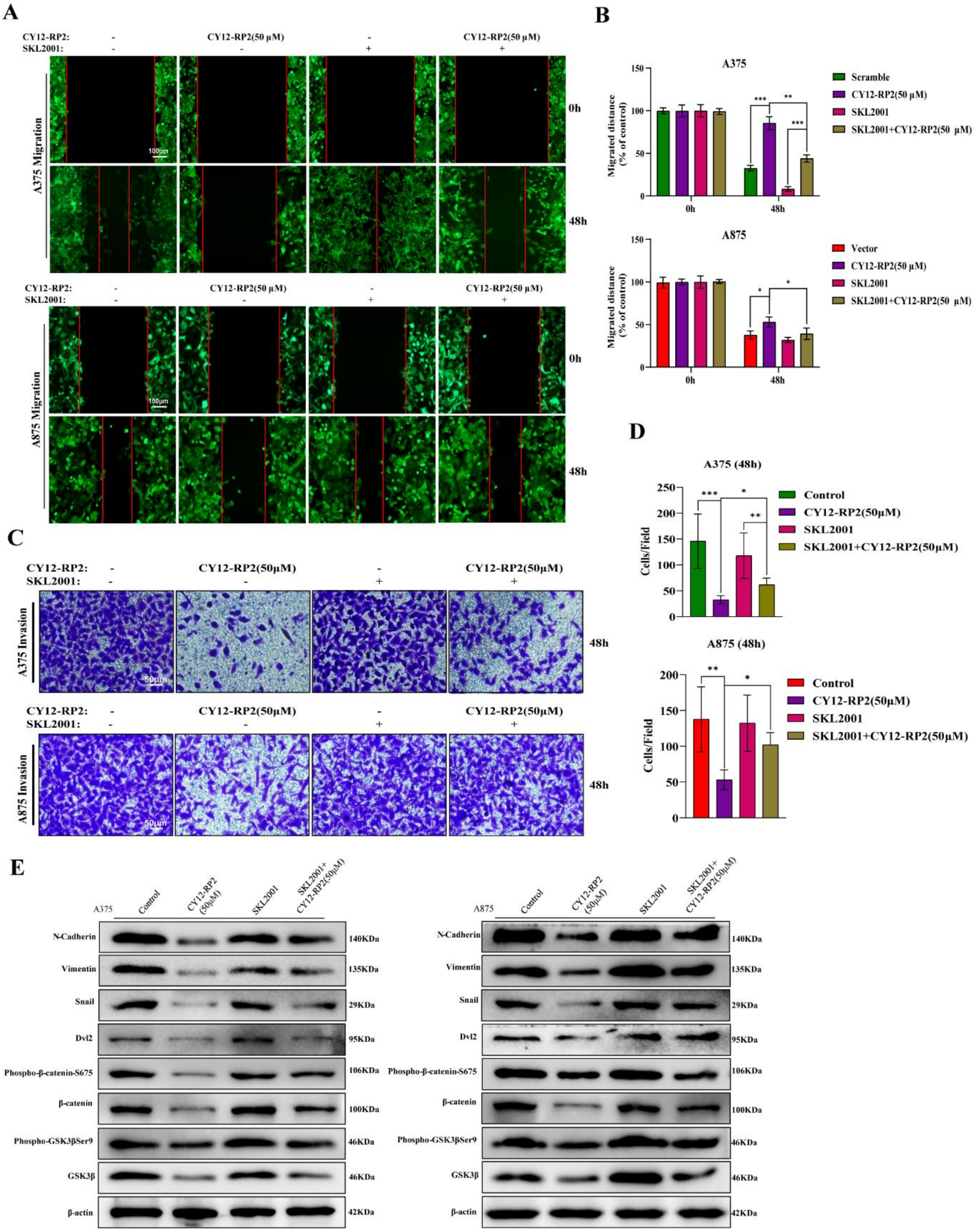
A
CY12-RP2: - CY12-RP2(50 μM) - CY12-RP2(50 μM)
SKL2001: - - + +
A375 Migration
A875 Migration
0h
48h
100μm
B
A375
A875
Migrated distance (% of control)
Scramble
Vector
CY12-RP2(50 μM)
SKL2001
SKL2001+CY12-RP2(50 μM)
C
CY12-RP2: - CY12-RP2(50μM) - CY12-RP2(50μM)
SKL2001: - - + +
A375 Invasion
A875 Invasion
50μm
D
A375 (48h)
A875 (48h)
Cells/Field
Control
CY12-RP2(50μM)
SKL2001
SKL2001+CY12-RP2(50μM)
E
A375
A875
Control
CY12-RP2 (50μM)
SKL2001
SKL2001+ CY12-RP2(50μM)
N-Cadherin 140KDa
Vimentin 135KDa
Snail 29KDa
Dvl2 95KDa
Phospho-β-catenin-S675 106KDa
β-catenin 100KDa
Phospho-GSK3βSer9 46KDa
GSK3β 46KDa
β-actin 42KDa

Fig. S5

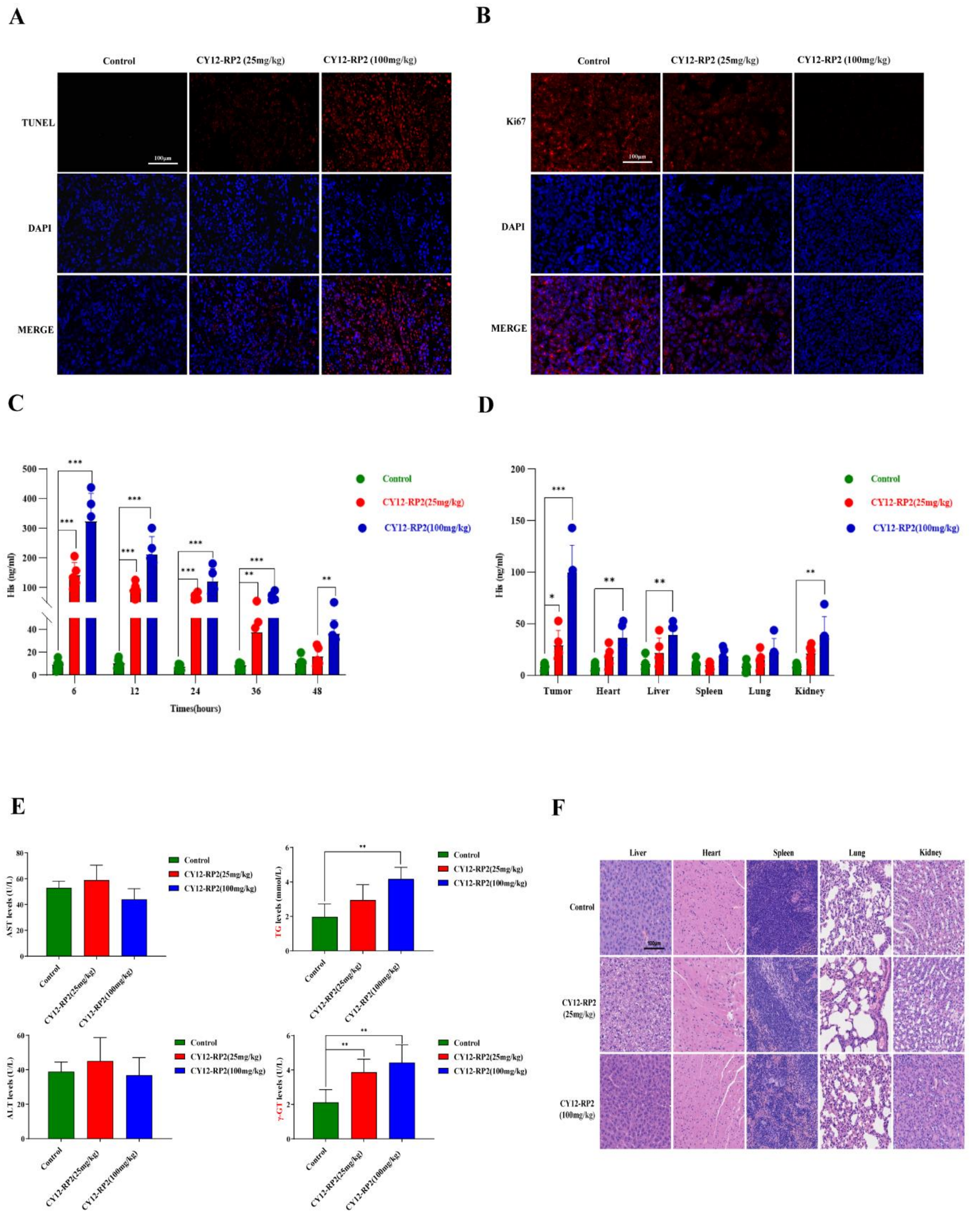
A
Control
CY12-RP2 (25mg/kg)
CY12-RP2 (100mg/kg)
TUNEL
DAPI
MERGE
100μm
B
Control
CY12-RP2 (25mg/kg)
CY12-RP2 (100mg/kg)
Ki67
DAPI
MERGE
100μm
C
His (ng/ml)
Times(hours)
6
12
24
36
48
***
**
Control
CY12-RP2(25mg/kg)
CY12-RP2(100mg/kg)
D
His (ng/ml)
Tumor
Heart
Liver
Spleen
Lung
Kidney
***
**
*
Control
CY12-RP2(25mg/kg)
CY12-RP2(100mg/kg)
E
AST levels (U/L)
ALT levels (U/L)
TG levels (mmol/L)
γ-GT levels (U/L)
Control
CY12-RP2(25mg/kg)
CY12-RP2(100mg/kg)
**
F
Liver
Heart
Spleen
Lung
Kidney
Control
CY12-RP2 (25mg/kg)
CY12-RP2 (100mg/kg)
100μm

Fig. S6

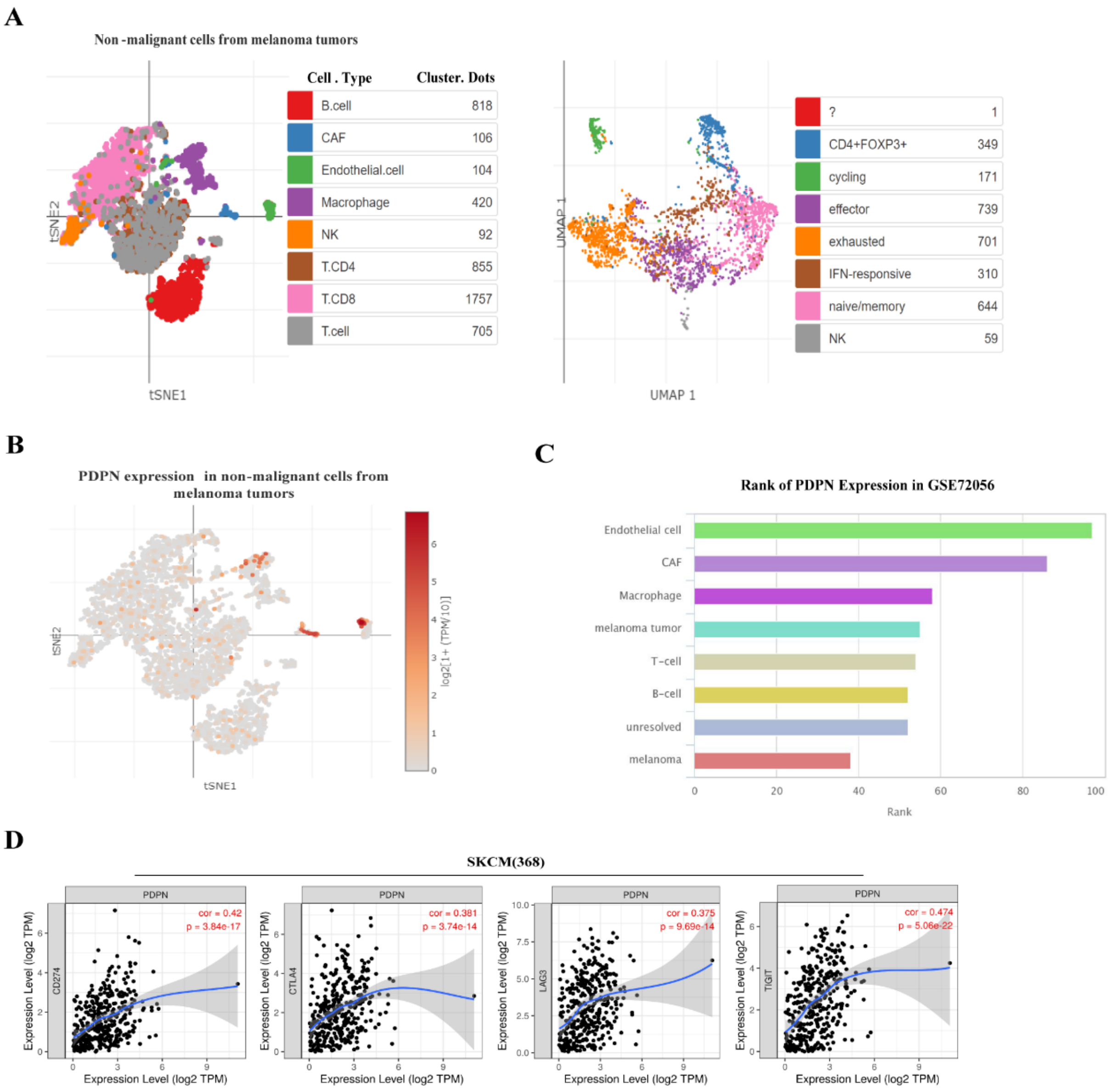

E

Cancer	CD8+ T cells	CD4+ T cells	M1 Macrophage	M2 Macrophage	Treg cells	NK cells
SKCM (n=471)	-2.988	-0.209	-5.524	6.865	1.457	-2.557
SKCM -Metastasis (n=368)	-2.464	0.03	-5.512	6.32	1.582	-2.161
SKCM -Primary (n=103)	-1.488	-0.05	-0.563	1.691	-0.768	-0.468

Fig. S7

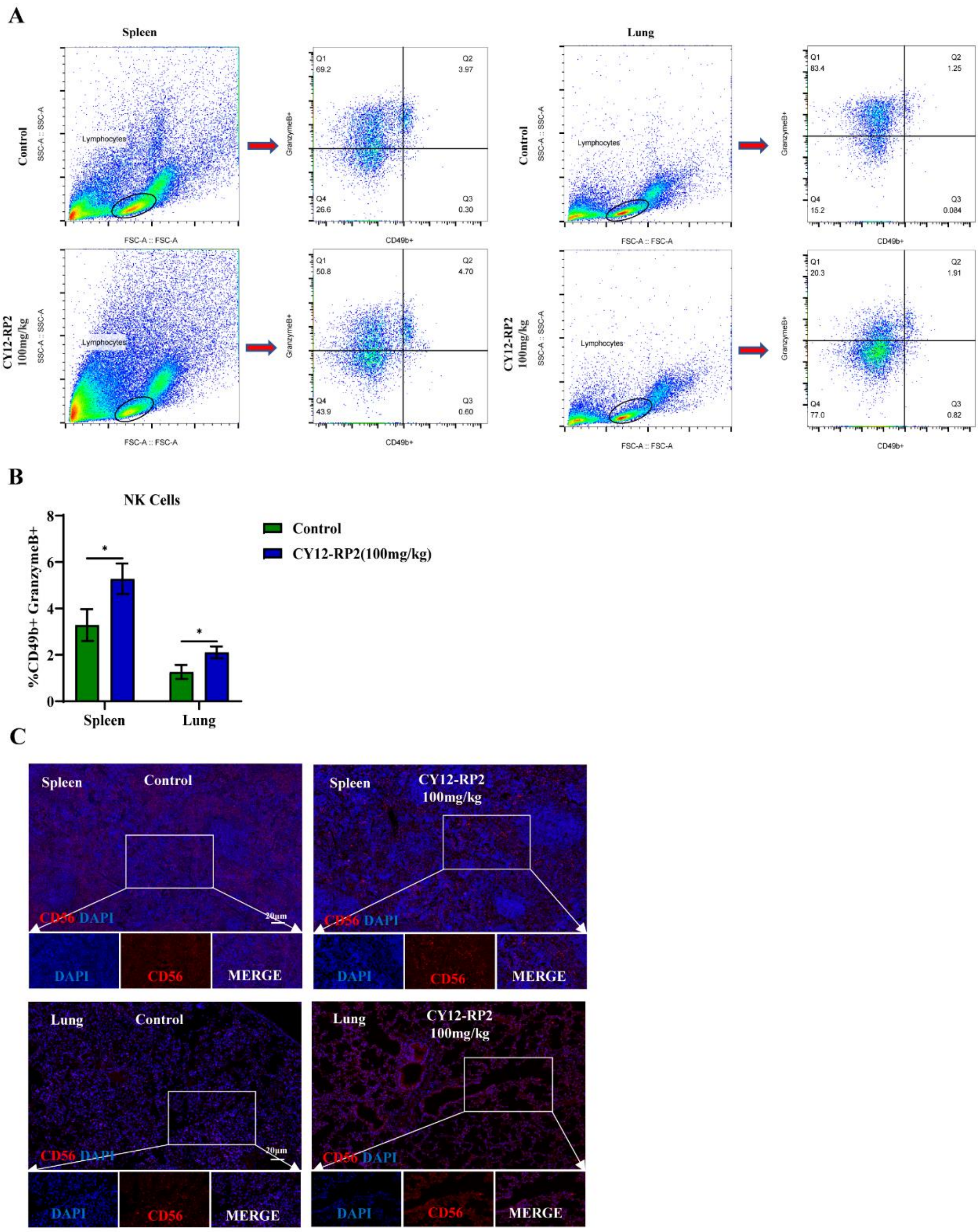

A
Spleen
Lung
Control
CY12-RP2 100mg/kg
Lymphocytes
SSC-A :: SSC-A
FSC-A :: FSC-A
GranzymeB+
CD49b+
Q1 69.2
Q2 3.97
Q4 26.6
Q3 0.30
Q1 50.8
Q2 4.70
Q4 43.9
Q3 0.60
Q1 83.4
Q2 1.25
Q4 15.2
Q3 0.084
Q1 20.3
Q2 1.91
Q4 77.0
Q3 0.82
B
NK Cells
%CD49b+ GranzymeB+
Control
CY12-RP2(100mg/kg)
Spleen
Lung
C
Spleen
Lung
Control
CY12-RP2 100mg/kg
CD56 DAPI
20μm
DAPI
CD56
MERGE

Fig. S8

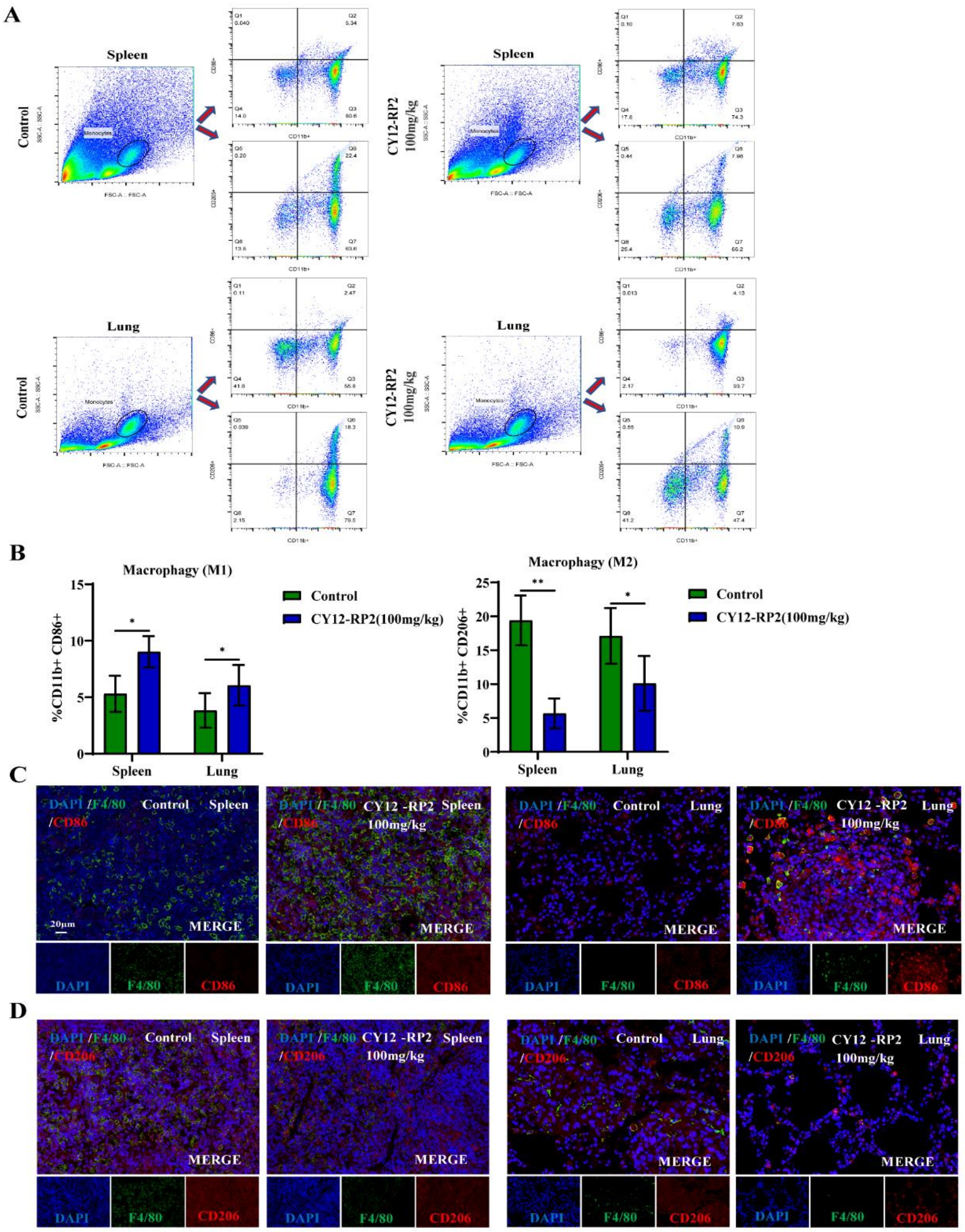
A
Spleen
Control
CY12-RP2 100mg/kg
Lung
B
Macrophagy (M1)
Macrophagy (M2)
Control
CY12-RP2(100mg/kg)
%CD11b+ CD86+
%CD11b+ CD206+
Spleen
Lung
C
D
DAPI /F4/80 /CD86
DAPI /F4/80 /CD206
MERGE
20μm
DAPI
F4/80
CD86
CD206

Fig. S1: PDPN was upregulated in melanoma, which was prognostically relevant to melanoma patient survival

(A) PDPN was significantly associated with the development of several types of tumors from the TCGA database. **(B)** Relationship between PDPN expression and prognosis in melanoma. **(C)** The mRNA expression of PDPN in melanoma cell lines (A375, SK-MEL-28, A875) were measured by real-time RT-PCR. **(D)** The protein levels of PDPN in melanoma cell lines (A375, SK-MEL-28, A875) were measured by western blot. **(E)** Immunofluorescence staining of PDPN in melanoma cell lines (A375, A875). The power field scale bar, 20 μm and 5μm. **(F)** The interference and overexpression efficiency of PDPN were determined by western blot (F). **(G)** The mRNA levels of c-MYC, Cyclin D1 and TWIST in A375 cells stably knockdown PDPN or A875 cells with PDPN overexpression. **(H, I)** Western blot assay was performed on VE-Cadherin, c-MYC, Cyclin D1 and TWIST in the PDPN knockdown A375 cells or in PDPN overexpression A875 cells, respectively (H). Statistical analysis was performed to quantify the relative protein levels (I). Data are presented as mean ± SD. *$p < 0.05$, **$p < 0.01$, ***$p < 0.001$.

Fig. S2: The effects of PDPN on migration and invasion could be modulated by SKL2001and IWR1

(A, B) Effects of PDPN on the migration ability of melanoma cells was detected by wound healing assays using SKL2001 and IWR1(A). Statistical analysis was performed to determine the migrated distance (B). The power field scale bar, 100 μm. **(C, D)**

Transwell assays were performed to quantify the modulatory effects of SKL2001 and IWR1 (C). Statistical analysis was performed to evaluate the invasion of cells (D). The power field scale bar, 100 μm. **(E)** Western blot assays were also performed on EMT-related proteins to identify the effects of SKL2001 and IWR1. Data are presented as mean ± SD. *p < 0.05, **p < 0.01, ***p< 0.001 versus control.

Fig. S3: The identification and functional analysis of PDPN- binding peptides

(A) Cell viability of melanoma cell lines (A375, A875) treated with 6 peptides except CY12-RP2 at various concentrations for 48 h. **(B)** Colocalization of CY12-RP1 and PDPN protein was performed by immunofluorescence in melanoma cell lines (A375, A875). The power field scale bar, 20 μm. **(C)** SPR assay was performed to quantify the binding activity of CY12-RP1 (100 μM, 20 μM, 4.0 μM, 0.8 μM, 0.16 μM, 0.032 μM, 0.0064 μM, 0.00128 μM) to PDPN. **(D)** A representative flow cytometry analysis showed the binding efficiency of CY12-RP2 with PDPN at various concentrations in melanoma cell lines (A375, A875). **(E)** High-performance liquid chromatography (HPLC) and mass spectrometry (MS) were performed to confirmed the purity of CY12-RP2. **(F)** Assessing the anti-tumor effects of CY12-RP2 on the viability of melanoma cell spheroids by using cell-processing system, and representative images of 2D cells and 3D cellular sphere were shown. The power field scale bar, 50 μm and 100 μm. **(G)** Immunofluorescence staining was performed to measure the caspase3 fluorescence intensity of A375 cell spheroids after treating with CY12-RP2. The power field scale bar, 100 μm.

Fig. S4: The inhibition of cells metastasis and invasion by CY12-RP2 can be reversed by SKL2001

(A, B) The inhibition of CY12-RP2 on the migration capacity of melanoma cell lines was reversed by SKL2001 (A). Statistical analysis was performed to determine the migrated distance (B). The power field scale bar, 100 μm. **(C, D)** Effect of CY12-RP2 on the invasive ability of melanoma cell lines were reversed by SKL2001 (C). Statistical analysis was performed to count the invaded cells (D). The power field scale bar, 100 μm. **(E)** Western blot assay was also performed to detect the proteins related to EMT and Wnt/β-catenin signaling pathway, including N-cadherin, Vimentin, snail, Dvl2, Phospho-β-catenin-S675, β-catenin, Phospho-GSK3β-Ser9 and GSK3β by CY12-RP2 with or without SKL2001 (50 μM). Data are presented as mean ± SD. $*p < 0.05$, $**p < 0.01$, $***p < 0.001$ versus control.

Fig. S5: Effects and toxicity of CY12-RP2 in vivo

(A) TUNEL analysis of apoptosis was conducted on orthotopic xenografts tumor sections using fluorescence staining. The power field scale bar, 100 μm. **(B)** Fluorescence staining of Ki67 was carried out on orthotopic xenograft tumor sections. The power field scale bar, 100 μm. **(C)** Serum His-CY12-RP2 levels of the orthotopic xenografts mice treated by various dosage of CY12-RP2 were measured by His-ELISA kit. **(D)** Tumor, heart, liver, spleen, lung, kidney His-CY12-RP2 levels of the orthotopic xenografts mice treated by various dosage of CY12-RP2 were measured by His-ELISA kit. **(E)** Serum ALT, AST, TG, and γ-GT levels of the orthotopic xenografts model were measured by ELISA. **(F)** H&E staining of liver, heart, spleen, lung, and kidney treated

with CY12-RP2 (25 mg/kg, 100 mg/kg) daily for 15 days. The power field scale bar, 100 μm. Data are presented as mean ± SD. *p < 0.05, **p < 0.01, ***p< 0.001 versus control.

Fig. S6: The expression of PDPN is associated with immune infiltration of melanoma tumors

(A, B) Single-cell RNA-seq of melanoma immunotherapy outcomes elucidated the role of PDPN related to the proportion of immune cells in the tumor microenvironment. Immune cell populations from melanoma tumors (A). PDPN expression in immune cells, notably T cells and macrophages (B). **(C)** Rank of PDPN expression in GSE72056 from TCGA database. **(D)** The expression correlation between PDPN and immunoinhibitory in SKCM (skin cutaneous melanoma) was investigated using the TISIDB database. **(E)** The correlation of PDPN expression with immune infiltration level in SKCM was investigated using the TIMER database.

Fig. S7: CY12-RP2 modifies the population of NK cells in the spleen and lung of mice

(A) Flow cytometric analysis of CD49b+ and Granzyme B+ populations in spleen and lung of BALB/C mice with lung metastases. **(B)** Statistical analysis was performed to count the percentage of CD49b+ and Granzyme B+ NK cells in the spleen and lung of BALB/C mice with lung metastases. **(C)** Fluorescence imaging of NK cells in the spleen and lung of pulmonary metastasis model stained with CD56 antibody. The power

field scale bar, 20 μm. Data are presented as mean ± SD. *$p < 0.05$, **$p < 0.01$, ***$p< 0.001$ versus control.

Fig. S8: The effects of CY12-RP2 on the proportion of macrophages (M1 and M2) in the spleen and lung of mice

(A) Representative flow cytometry images showing the proportion of M1 and M2 macrophages in the spleen and lung of BALB/C mice with lung metastases. **(B)** Statistical analysis was performed to count the percentage of CD11b+ CD86+ M1 macrophages and CD11b+ CD206+ M2 macrophages in the spleen and lung of BALB/C mice with lung metastases. **(C, D)** Proportion of M1 (F4/80+ CD86+) macrophages (C) and M2 (F4/80+ CD206+) macrophages (D) in the spleen and lung of BALB/C mice with lung metastases were detected by representative double immunofluorescence. The power field scale bar, 20 μm. Data are presented as mean ± SD. *$p < 0.05$, **$p < 0.01$, ***$p< 0.001$ versus control.

Table 1: Antibodies applied for western-blotting, flow cytometry, immunofluorescence and immunohistochemistry analysis

antibodies	sources	catalog No
mouse anti-human pdpn/gp36	BioLegend	127407
Rabbit anti-human pdpn/gp36	ABclonal	A9242
Rabbit anti-human N-Cadherin	ABclonal	A19083
mouse anti-human E-cadherin	ABclonal	A18135
Rabbit anti-human Vimentin	ABclonal	A11952
Rabbit anti-human snail	Abclonal	A5243
Rabbit anti-human Axin1	CST	#2087
Rabbit anti-human Dvl2	CST	#3224
Rabbit anti-human Phospho-β-catenin-S675	ABclonal	AP0795
Rabbit anti-human β-catenin	ABclonal	A0316
Rabbit anti-human Naked1	CST	#2262
Rabbit anti-human Phospho-GSK3β-Ser9	ABclonal	AP0039
Rabbit anti-human GSK3β	ABclonal	A6164
Rabbit anti-human LEF1	ABclonal	A4473
Rabbit anti-human TCF1/TCF7	ABclonal	A20835
Rabbit anti-human Caspase3	ABclonal	A2156
Rabbit anti-human Caspase9	ABclonal	A2636
Rabbit anti-human BCL2	ABclonal	A111025
Rabbit anti-human Bax	ABclonal	A0207
mouse anti-human β-actin	ABclonal	AC004
FITC anti-mouse CD3	Biolegend	100203
PE-Cyanine7 Anti-Mouse CD4	Tonbo	60-0042-U025
PerCP-Cyanine5.5 Anti-Mouse CD8a	Tonbo	65-0081-U025
APC anti-mouse CD25	Biolegend	102011
PE Anti-Mouse Foxp3	Tonbo	50-5773-U025
APC/Cyanine7 anti-mouse CD49b	Biolegend	108919
PE anti-human/mouse Granzyme B	Biolegend	372207
APC anti-mouse/human CD11b	Biolegend	101211
FITC anti-mouse CD86	Biolegend	105006
PE anti-mouse CD206	Biolegend	141705
